# Insights into factors governing encapsulation and release performance of starch-based nanocarriers for nutraceuticals delivery

**DOI:** 10.3389/fnut.2026.1816871

**Published:** 2026-04-29

**Authors:** Youming Zuo, Iris J. Joye

**Affiliations:** Department of Food Science, University of Guelph, Guelph, ON, Canada

**Keywords:** bioactive compound, encapsulation efficiency, nutraceutical, release performance, starch-based nanocarrier

## Abstract

Starch-based nanocarriers offer a versatile platform for enhancing the stability and bioavailability of labile nutraceuticals. Their performance, however, is highly sensitive to complex interacting variables, constraining rational design. This review systematically analyzes the critical factors governing encapsulation efficiency and release kinetics to establish factor–property–function correlations. Key findings reveal that carrier performance is not determined by a single variable but by the synergistic interplay of colloidal properties (particle size, PDI, zeta potential), material structure (crystallinity, amylose/amylopectin ratio), and preparation methods. Furthermore, the intrinsic properties of bioactive compounds (solubility, size, charge) are identified as primary drivers that dictate the binding and retention mechanisms. Consequently, a bioactive-driven rational design strategy is proposed, suggesting that carrier type should be precisely tailored to the specific cargo. This review provides essential guidance for the development of high-performance starch-based delivery systems for nutraceuticals. Future research should focus on precise structural modulation and performance verification within complex food matrices to bridge the gap between laboratory design and industrial application.

## 1 Introduction

Nutraceuticals are bioactive compounds derived from food sources that confer both nutritional and therapeutic benefits, bridging the gap between conventional nutrition and pharmacology. They encompass diverse molecules: polyphenols, carotenoids, omega-3 fatty acids, probiotics, and phytochemicals, which are known to modulate oxidative stress, inflammation, and metabolic regulation (1). In recent years, their role in disease prevention and health optimization has gained increasing recognition, fueling a global market projected to surpass USD 650 billion by 2027, driven by the “food as medicine” paradigm (2). However, their clinical potential remains constrained by key physicochemical barriers, including low water solubility, instability under physiological conditions, and poor intestinal absorption, which collectively lead to limited bioavailability and inconsistent therapeutic efficacy (3). These challenges have accelerated the adoption of nanotechnology-based delivery systems inspired by pharmaceutical sciences. Nanoencapsulation and nanocarrier technologies, such as liposomes, solid lipid nanoparticles, polymeric micelles, and nanoemulsions, provide enhanced stability, protection from degradation, and controlled or targeted release of nutraceuticals (4, 5). Lipid-based nanoparticles, for instance, have shown notable efficacy in improving the stability and bioavailability of lipophilic compounds such as curcumin, resveratrol, and carotenoids (6). Moreover, recent advances in nanodelivery platforms, including bilosomes, nanomicelles, and hybrid emulsions, offer improved intestinal uptake and bioaccessibility of encapsulated compounds, bridging the long-standing bioavailability gap between nutraceuticals and pharmaceuticals (7, 8). Collectively, these developments establish nanodelivery as a transformative approach to enhancing the bioavailability performance, safety, and efficacy of nutraceuticals, positioning nanonutraceuticals as the next frontier in functional food and therapeutic innovation (9).

Starch is a naturally abundant, biodegradable polysaccharide composed primarily of two glucose polymers: amylose, a mostly linear molecule with α-(1 → 4) linkages, and amylopectin, a highly branched molecule with α-(1 → 4) and α-(1 → 6) linkages. It can be obtained from various botanical sources such as corn, potato, cassava, and rice, and serves as a renewable and biocompatible material for food, pharmaceutical, and biomedical applications (10). Due to its non-toxic nature, chemical versatility, and functional modifiability, starch has become an attractive biopolymer for developing nanocarrier systems. Its abundance, low cost, and ability to form stable inclusion complexes or nanostructures make it particularly suitable for encapsulating and protecting sensitive bioactive compounds in food applications (11, 12). Starch-based nanocarriers offer a sustainable alternative to synthetic polymers by combining biodegradability, hydrophilicity, and tunable physicochemical properties essential for controlled release and targeted delivery of nutraceuticals (13). Their versatility stems from starch’s hierarchical structure and ability to form nanoscale architectures with tunable functionality. Starch nanoparticles, nanocrystals, and nanospheres are typically obtained through physical, chemical, or enzymatic modification methods such as acid hydrolysis or nanoprecipitation, producing nanostructures with large surface areas, reactive hydroxyl groups, and adjustable crystallinity that favor the adsorption and controlled release of bioactives (14, 15). These nanostructures can act as both carriers and stabilizers in emulsion-based delivery systems, particularly in Pickering emulsions, where starch nanocrystals or nanoparticles adsorb at oil–water interfaces to improve the stability and bioaccessibility of encapsulated lipophilic nutraceuticals (16, 17). Meanwhile, amylose-guest inclusion complexes (V-type complexes) are formed when linear amylose helices encapsulate small hydrophobic molecules through cooperative hydrophobic interactions and hydrogen bonding, resulting in V-type crystalline structures that enhance the dispersibility oxidative stability, and thermal resistance of incorporated bioactives (18, 19). Beyond particulate carriers, starch-based nanogels provide soft, stimuli-responsive networks capable of encapsulating both hydrophilic and hydrophobic compounds and releasing them in response to thermal, pH or enzymatic triggers (20, 21). Similarly, starch-biopolymer composites, formed by blending starch with proteins, lipids, or other polysaccharides, yield hybrid nanomaterials in which the complementary functionalities of each component collectively enhance barrier, mechanical, and encapsulation performance, supporting multifunctional applications in nutraceutical and food systems (22). Starch-based nanocarriers effectively enhance the solubility, stability, and controlled release of encapsulated bioactive compounds, overcoming challenges of poor bioavailability and degradation in harsh gastrointestinal environments (23). Their pH-sensitive and enzyme-responsive degradation enables targeted release that aligns with physiological digestion processes, ensuring efficient intestinal absorption of nutraceuticals (24). Overall, starch-based nanocarriers provide a biocompatible and versatile platform for nutraceutical delivery, combining the inherent functionality of natural polymers with the structural precision of nanotechnology.

The evolution of drug delivery systems has profoundly influenced the development of nutraceutical delivery technologies (25). Concepts such as targeted transport, stimuli-responsive degradation, and controlled release initially designed for pharmaceuticals, now guide the design of starch-based nanocarriers for bioactive compounds (26). From a food and nutraceutical perspective, the functionality of starch-based nanocarriers must be evaluated in the context of both food processing and gastrointestinal digestion, rather than solely be based on encapsulation performance under idealized conditions (27).

Effective nutraceutical delivery systems must maintain structural stability during processing and storage to prevent premature release or degradation prior to consumption. During food processing, starch-based nanocarriers can protect bioactive compounds by encapsulation, isolating them from unfavorable external environments. This reduces the risk of degradation caused by common processing stresses such as high temperature, extreme pH, and mechanical shear, while also limiting stability losses during storage due to exposure to oxygen and light, moisture fluctuations, and microbial contamination (28). Following ingestion, starch-based nanocarriers encounter highly dynamic gastrointestinal environments characterized by enzymatic hydrolysis, bile salt interactions, and sequential pH transitions, which collectively govern carrier disassembly and bioactive release behavior (29). In food systems, therefore, “controlled release” does not simply imply sustained release, but rather the ability to withstand food processing while enabling site-appropriate release during digestion, typically favoring release in the small intestine where absorption occurs (30).

Understanding how these material–structure–function relationships operate across both processing and digestive stages is essential for tailoring encapsulation systems to specific nutraceuticals and for translating nanostructure design into meaningful improvements in bioaccessibility and bioavailability (31). However, most current reviews emphasize the application of starch-based nanocarriers rather than dissecting the factors that drive their encapsulation and release dynamics. This creates a critical knowledge gap between material science and functional performance optimization. Importantly, these governing factors do not operate independently, because colloidal properties, structural organization, preparation conditions, and the physicochemical characteristics of bioactive compounds are closely interconnected in determining encapsulation efficiency, stability, and release behavior. A change in one variable may therefore alter the functional contribution of the others. To provide a transparent basis for this discussion, the literature considered in this review was identified through structured searches of Google Scholar, Scopus, PubMed, and Web of Science using combined keywords related to starch materials, nanoscale carrier systems, encapsulation and release behavior, and bioactive compounds. Studies focused primarily on Pickering emulsions were excluded. Priority was given to recent peer reviewed studies, while a limited number of older foundational studies were included when they provided representative insights that remain important to the field. Therefore, this review provides a comprehensive synthesis and discussion of the key factors influencing the performance of starch-based nanocarriers, including the intrinsic properties of starch, nanocarrier fabrication methods, characteristics of encapsulated bioactives, and types of starch-based nanocarriers. By clarifying these relationships, this review aims to bridge the gap between material design and delivery performance, offering theoretical and practical insights to guide the rational development of starch-based delivery systems for nutraceutical and functional food applications.

## 2 Intrinsic material factors

### 2.1 Particle size, polydispersity index and zeta potential

The performance of starch-based nanocarriers in bioactive delivery is influenced by their physicochemical properties. Among these, particle size, polydispersity index (PDI) and zeta potential (*ζ*-potential) are regarded as the core parameters determining carrier behavior. These three indicators directly reflect the structural scale, distribution uniformity, and colloidal stability of the nanosystem, serving as primary factors influencing the loading capacity of active ingredients, dispersion behavior, storage stability, and release kinetics. Given that starch itself possesses inherent characteristics such as swellability, degradability, and chemical modifiability, the particle size, charge, and dispersion uniformity often undergo significant changes depending on the preparation method, environmental conditions, and substrate interactions (32–34). Therefore, before delving into other factors, it is essential to first clarify the definitions, significance, and functional roles of these parameters within the delivery system.

Particle size refers to the typical diameter or dimension of the particle (usually measured in nanometers), which ranges from tens to hundreds of nanometers in the field of nanocarriers. For starch-based nanoparticles, this dimension determines the surface area, diffusion path, and capacity for interaction with the biological system, thereby playing a central role in encapsulation and release mechanisms (35, 36). Smaller particles typically provide a higher specific surface area, creating more contact interfaces between the carrier and the bioactive substance. This facilitates loading and interfacial adsorption while shortening the diffusion distance, resulting in faster and more controlled release rates. Conversely, larger particles may exhibit slower release profiles when diffusion-controlled transport dominates, owing to longer diffusion pathways. Particle size also plays a key role in governing carrier stability in the gastrointestinal tract and modulating cellular uptake pathways, thereby influencing overall bioavailability (37–40).

PDI reflects the degree of uniformity in particle size distribution. It modulates the delivery behavior of starch-based nanocarriers by affecting system stability, loading uniformity, and the predictability of release. When the PDI is low, all particles exhibit near-consistent dimensions, implying that the entire carrier system possesses uniform porosity, surface area, and diffusion paths during the loading of bioactive substances. Consequently, the system demonstrates almost synchronous kinetic behavior during the release process, making the release profile more stable and easier to model. However, when the PDI is high, the system simultaneously contains large particles (prone to sedimentation, slow release, and poor adsorption capacity) and small particles (which may lead to burst release). This heterogeneity results in uneven encapsulation efficiency, chaotic release patterns, and reduced efficiency in reaching the target site. Furthermore, systems with excessive PDI are more susceptible to aggregation or phase separation during food processing and storage, causing the premature release or degradation of bioactive substances. Therefore, researchers generally consider PDI a necessary quality parameter for constructing controllable delivery systems (41, 42).

*ζ*-potential is the electrokinetic potential at the slipping plane of particles in dispersion, reflecting the combined effects of surface charge and associated counter-ions in the surrounding electrical double layer. It influences bioactive delivery by modulating particle-particle repulsion, interfacial adsorption of biomolecules onto the particle surface, and carrier behavior within the gastrointestinal tract. A high absolute value of *ζ*-potential (typically > 30 mV) signifies significant electrostatic repulsion, which keeps particles dispersed and prevents aggregation or sedimentation, thereby maintaining the encapsulation stability of bioactives and avoiding non-targeted release caused by aggregation. If the absolute value of the *ζ*-potential is low, the system is prone to flocculation, rendering the release behavior uncontrollable. Additionally, surface charge modulates the binding energy between the bioactive substance and the starch matrix (36, 41). Moreover, in the gastrointestinal environment, charge influences particle integrity, and the adsorption behavior of the carrier onto the mucus layer and cell membranes, thereby altering bioavailability (43, 44). Thus, a favorable and controllable *ζ*-potential ensures not only dispersion stability but also influences binding strength, diffusion rate, and targeted release.

Table 1 summarizes the influence of colloidal properties of starch-based nanocarriers on their encapsulation, release, and stability performance for nutraceutical delivery in recent studies. It is crucial to recognize that particle size, PDI, and *ζ*-potential are not mutually independent characterization dimensions within starch-based nanocarrier systems. Instead, these three parameters collectively determine the dispersion stability of particles in the aqueous phase, the available specific surface area of the nanoparticles, the binding mode of bioactive substances, and the subsequent release kinetics. Starch molecular chains contain abundant hydroxyl groups, making them prone to forming intramolecular and intermolecular hydrogen bonds (45–47). These interactions complicate chain aggregation and rearrangement during particle formation, making particle size difficult to control and often leading to the coexistence of distinct particle populations in the aqueous phase. This heterogeneity leads to an increase in PDI, thereby undermining the electrostatic repulsion that would otherwise be provided by the *ζ*-potential. As a result, starch-based particles become more susceptible to hydrogen bond bridging or local aggregation, leading to a risk of sedimentation within the suspension system. Conversely, when the particle size distribution of starch-based nanocarriers is narrower, the system exhibits a lower PDI. Under these conditions, a more uniform surface charge distribution enhances electrostatic repulsion, as reflected by the *ζ*-potential, thereby reducing particle aggregation and improving colloidal stability. Furthermore, in starch systems, *ζ*-potential can explicitly exert a reciprocal influence on particle size and PDI. A higher absolute *ζ*-potential value can inhibit the secondary aggregation of small particles during processes such as gelatinization-redispersion, acid hydrolysis, nanoprecipitation, or redispersion after drying, thereby ensuring the generated starch-based nanoparticles maintain a smaller size and lower PDI. If the *ζ*-potential is low and electrostatic repulsion is insufficient, starch chain segments tend to aggregate via hydrogen bonding or hydrophobic domains (if present), ultimately forming larger particles and significantly broadening the PDI.

**Table 1:** Recently reported colloidal properties and encapsulation, release, and stability performance of starch-based nanocarriers for nutraceutical delivery.

Starch type	Encapsulated nutraceuticals	Colloid properties	Encapsulation, release, and stability performance	References
Cassava starch	Propolis polyphenols	Particle size (159–182 nm), PDI (0.18–0.25)	Improved thermal and pH stability with smaller particle size and lower PDI	(130)
Cassava starch	Curcumin	Particle size (167–211 nm), PDI (0.19–0.27)	Higher LE (85% → 91%) and LC (1.12 → 1.54 mg/g), and more sustained release with larger particle size	(56)
Debranched waxy maize starch	Curcumin	Particle size (970–1,208 nm), PDI (0.24–0.33), *ζ*-potential (−14 to −42 mV)	Higher EE (54% → 63%) and more sustained release with smaller particle size and higher absolute *ζ*-potential	(114)
Debranched waxy maize starch	Curcumin	Particle size (100–>1,000 nm), PDI (<0.30–0.344)	Higher EE (50–56% → 82–86%) and more sustained release with smaller particle size and lower PDI	(131)
Debranched waxy maize starch	Curcumin	Particle size (178–124 nm), PDI (0.23–0.32), ζ-potential (−18 to −25 mV)	Improved dispersion stability and negligible size/PDI increase over 15 days of storage with smaller particle size and lower PDI	(132)
Debranched waxy maize starch	Curcumin	Particle size (139–329 nm), PDI (0.12–0.31), ζ-potential (−22.4 to −33.6 mV)	Higher EE (59% → 92%) and LC (19% → 27%) with larger particle size	(133)
Turmeric starch	Curcumin	Particle size (260–325 nm), PDI (0.23–0.44), ζ-potential (−10 to −13 mV)	Higher LE (43% → 59%) and LC (5 → 7 μg/mg), and improved thermal/ionic/pH/storage stability with smaller particle size and lower PDI	(134)
High amylose corn starch, potato starch	Curcumin	Particle size (32–99 nm), PDI (0.17–0.30), ζ-potential (−18 to −32 mV)	Higher EE (22% → 95%) with larger particle size, lower PDI, and higher absolute ζ-potential	(52)
Hydroxyethyl starch	Curcumin	Particle size (69–80 nm), PDI (0.13–0.13), ζ-potential (−27 to −28 mV)	More sustained release with larger particle size	(135)
Carboxymethylated short-chain amylose	Curcumin	Particle size (121–233 nm), PDI (0.13–0.42), ζ-potential (+27 to −33 mV)	Higher EE (34% → 97%), and more sustained gastrointestinal release with smaller particle size, lower PDI, and higher absolute ζ-potential	(136)
OSA starch	Curcumin	Particle size (129–272 nm), PDI (0.13–0.21), ζ-potential (−15 to −20 mV)	Faster dissolution and release with smaller particle size, lower PDI, and higher absolute ζ-potential	(137)
OSA starch	Curcumin	Particle size (14–40 nm), PDI (0.38–0.45), ζ-potential (−10 to −46 mV)	Higher EE (37% → 78%) and LC (0.5% → 1.6%) with smaller particle size and higher absolute ζ-potential	(115)
OSA starch	Curcumin	Particle size (164–210 nm), PDI (0.08–0.12), ζ-potential (+26 to +1 mV)	Higher EE (56% → 89%) and LC (15% → 24%), and improved storage stability with smaller particle size and higher absolute ζ-potential	(116)
Debranched cassava starch	Curcumin	Particle size (91–154 nm), PDI (0.22–0.38)	Lower LE (66% → 30%) and LC (22 → 11 mg/g), improved dispersion and stability, and more controlled gastric release with smaller particle size and lower PDI	(138)
Waxy corn starch	Curcumin	Particle size (180–260 nm), PDI (0.12–0.40)	Higher LE (33% → 55%) and LC (1.6 → 2.8%), and improved colloidal stability with smaller particle size and lower PDI	(57)
Corn starch	Anthocyanins	Particle size (520–659 nm), PDI (0.50–0.55), ζ-potential (−5 to −35 mV)	More sustained release with higher absolute ζ-potential	(139)
Corn starch	Anthocyanins	Particle size (65–390 nm)	Higher EE (45% → 53%) with larger particle size	(53)
Soluble dextrin	Anthocyanins	Particle size (73–128 nm), PDI (0.29–0.31), ζ-potential (−39 to −40 mV)	Higher EE (88% → 97%), lower cumulative release after 24 h (16% → 11%), and improved storage stability with larger particle size, lower PDI, and higher absolute ζ-potential	(140)
Maize starch	Anthocyanins	Particle size (50–500 nm)	Improved stability and reduced release at higher pH with larger particle size	(141)
Potato amylopectin	Anthocyanins	Particle size (increased after anthocyanin binding)	More sustained release and improved oxidative stability with larger particle size after binding	(142)
Waxy maize starch	Catechin, epicatechin, epigallocatechin gallate, procyanidins	Particle size (TEM: 40–150 nm; DLS: 100–400 nm), ζ-potential (−10 to −28 mV)	Improved antioxidant stability with larger particle size and higher absolute ζ-potential	(143)
Horse chestnut starch, water chestnut starch, lotus stem starch	Catechin	Particle size (323–616 nm), PDI (0.20–0.62), ζ-potential (−18 to −20 mV)	Higher EE (48% → 59%) and entrapped catechin (4.83 → 5.92 mg/100 mg), more sustained release, and higher stability with larger particle size, higher PDI, and higher absolute ζ-potential	(144)
Quinoa starch, maize starch	Quercetin	Particle size (166–877 nm), PDI (0.43–0.72)	Higher LC (18% → 27%), improved storage stability, and more sustained antioxidant activity release with smaller particle size	(145)
*Ginkgo biloba* starch	Quercetin	Particle size (80–165 nm), PDI (0.28–0.34)	Sustained release with larger particle size and lower PDI	(146)
Debranched waxy maize starch	Quercetin	Particle size (201–276 nm), ζ-potential (+32 to −23 mV)	Higher EE (34% → 75%), slower release, and improved thermal stability with higher absolute ζ-potential	(147)
Debranched waxy maize starch	Resveratrol	Particle size (258–543 nm), ζ-potential (−13 to −18 mV)	Higher LC (7% → 20%) with smaller particle size and lower PDI	(148)
High amylose corn starch, potato starch	Vitamin D_3_	Particle size (14–40 nm), PDI (0.38–0.45), ζ-potential (−10 to −46 mV)	Higher EE (48% → 78%) and more sustained release with smaller particle size, lower PDI, and larger absolute ζ-potential	(149)
Corn starch	Vitamin D_3_	Particle size (DLS: <100–1,000 nm; AFM: 25–90 nm)	Improved stability and more sustained release with smaller particle size	(150)
Corn starch	Walnut peptide	Particle size (224–346 nm), PDI (0.39–0.61);	More sustained release and improved salt/pH/storage/serum stability with larger particle size	(151)
Corn starch	Apple polyphenols	Particle size (155–221 nm), ζ-potential (−6 to −26 mV)	Higher AC (2.68 → 37.02 mg/g), improved colloidal stability, and more sustained release with smaller particle size and higher absolute ζ-potential	(152)
Corn starch	Apple polyphenols	Particle size (221–338 nm), ζ-potential (−12 to −27 mV)	Improved antioxidant retention with larger particle size and higher absolute ζ-potential	(153)
Corn starch	Apple polyphenols	Particle size (221–419 nm), PDI (0.25–0.64)	Lower AC, more stable antioxidant activity, and more sustained intestinal release with smaller particle size and lower PDI	(154)
Sorghum starch, foxtail millet starch, pearl millet starch	β-carotene	Particle size (399–587 nm), ζ-potential (−18 to −22 mV)	Higher EE (78% → 90%) with smaller particle size and higher absolute ζ-potential; more sustained intestinal release with larger particle size	(58)
Maize starch	β-carotene	Particle size (<50–>200 nm)	Enhanced thermal stability with smaller particle size	(155)
Arrowhead starch, bracken starch, kudzu starch	Lactoferrin	Particle size (105–761 nm), PDI (0.33–0.70), ζ-potential (−6 to −15 mV)	Higher EE, improved colloidal stability and retention in GIF with smaller particle size, lower PDI, and higher absolute ζ-potential	(156)
Debranched quinoa starch, maize starch, waxy maize starch	Chrysin, rutin	Particle size (206–239 nm), PDI (0.23–0.39), ζ-potential (−10 to−21 mV)	Higher EE, enhanced stability, and more sustained release with smaller particle size and higher absolute ζ-potential	(157)
Lotus stem resistant starch	Kaempferol	Particle size (689–1,120 nm), PDI (0.57–0.75), ζ-potential (−21 to −22 mV)	EE maximum at intermediate particle size and moderate PDI	(158)
Lotus stem resistant starch	Crocin, kaempferol	Particle size (315–602 nm), PDI (0.23–0.85), ζ-potential (−24 to −28 mV)	Increased retention with larger particle size, lower PDI, and higher absolute ζ-potential	(159)
Debranched waxy maize starch	Sinigrin	Particle size (203–275 nm), ζ-potential (−20 to −30 mV)	Improved gastric stability and more sustained intestinal release with larger particle size and lower absolute ζ-potential	(160)
Waxy corn starch	*Eruca sativa* leaf polyphenolic extract	Particle size (50–268 nm), PDI (0.36–0.39), ζ-potential (−11 to −16 mV)	No significant change on EE (16.6% → 16.9%) with larger particle size, higher PDI, and lower absolute ζ-potential	(161)
Waxy maize starch	Resveratrol	Particle size (250–544 nm), PDI (0.20–bimodal)	Lower EE (79% → 37%) and LC (16% → 7%), more sustained release, and higher stability with smaller size and lower PDI	(162)
Lotus root starch	Luteolin	Particle size (280–502 nm), PDI (0.17–0.32), ζ-potential (−15 to −30 mV)	Higher EE (65% → 87%), more sustained intestinal release, and improved gastric stability with smaller particle size, lower PDI, and higher absolute ζ-potential	(163)
Sago starch	Piperine	Particle size (110–120 nm)	Higher LE (0.5% → 1.6%) and more sustained release with smaller particle size	(164)
Tapioca starch	Myricetin	Particle size (55–120 nm)	Higher AC (20 → 103 mg/g) and faster adsorption equilibrium with smaller particle size	(165)
Short amylose from corn	Rutin	Particle size (376–1,052 nm), ζ-potential (−9 to −26 mV)	Higher EE (80% → 86%) and more sustained release with larger particle size and lower absolute ζ-potential	(166)
Carboxymethyl starch	Rutin	Particle size (104–289 nm), PDI (0.13–0.45), ζ-potential (+32 to −35 mV)	Higher EE (52% → 74%), enhanced thermal /pH stability with smaller particle size, lower PDI, and higher absolute ζ-potential	(167)
*Agriophyllum squarrosum* starch	Lycopene	Particle size (562–616 nm), PDI (0.29–0.40), ζ-potential (−24 to −29 mV)	Lower EE (64% → 49%), higher antioxidant activity retention, and more sustained release with smaller particle size, lower PDI, and higher absolute ζ-potential	(168)
Debranched waxy maize starch	Tangeretin	Particle size (351–468 nm) PDI (0.18–0.32)	Higher (LA 17.04 → 17.91 μg/mg), LE (79% → 83%), and RI (93% → 99%) with larger particle size and lower PDI	(121)
Cassava starch	Propolis phenolic compounds	Particle size (24–614 nm); ζ-potential (−18 to −26 mV)	Lower LE (67% → 64%) with higher absolute ζ-potential	(169)
Glutinous rice starch	Phycocyanin	Particle size (357–463 nm), PDI (0.18–0.28), ζ-potential (−10 to −19 mV)	Higher EE (55% → 98%), LC (18% → 43%), and more sustained release with smaller particle size, lower PDI, and higher absolute ζ-potential	(122)
Debranched waxy maize starch	Tea polyphenols	Particle size (147–192 nm), ζ-potential (−9 to −22 mV)	Higher LC (26 → 47 μg/mg) and improved colloidal stability with larger particle size and higher absolute ζ-potential	(170)

Colloid properties are reported as ranges under different formulation, preparation, or bioactive incorporation conditions, as described in the original studies. The encapsulation, release, and stability performances reported reflect observed variations associated with corresponding colloidal properties (particle size, PDI, and ζ-potential). SNPs, starch nanoparticles; AC, adsorption capacity; LE, loading efficiency; EE, encapsulation efficiency; LC, loading capacity; RI, retention index; PDI, polydispersity index; ζ-potential, zeta potential; TEM, transmission electron microscopy; DLS, dynamic light scattering; AFM: atomic force microscopy; GIF, gastrointestinal fluids; OSA, octenyl succinic anhydride.

Therefore, particle size, PDI, and *ζ*-potential constitute a synergistic system within starch-based nanocarriers that can either mutually reinforce or weaken one another. Together, they regulate particle stability, the effectiveness of the specific surface area, and the diffusion paths of active ingredients within the starch chain network. For instance, even if the primary particle size is very small, if the PDI is high or the *ζ*-potential is inadequate, particles may still aggregate rapidly, causing the release profile to lose predictability. Conversely, if the PDI is low and the ζ-potential is high, even slightly larger particles can maintain controlled diffusion within the starch network. Given that most bioactive substances are prone to oxidation or degradation in the gastrointestinal environment, even minor fluctuations in these parameters can significantly alter encapsulation efficiency and release kinetics, and, hence, bioactive stability. Thus, they serve as the core variables for constructing high-performance starch-based nanodelivery systems.

### 2.2 Amylose/amylopectin ratio and crystallinity

Starch is composed of two major polysaccharides, amylose and amylopectin. In most botanical sources, amylose accounts for 20–30%, whereas amylopectin represents 70–80% of the total starch content (48–50). Amylose is primarily a linear or slightly branched polymer consisting of α-(1 → 4)-linked glucose units, which enables it to adopt flexible conformations in solution and readily form single-helical structures. These helices possess hydrophobic inner cavities that can accommodate small non-polar molecules, such as lipophilic vitamins, polyphenols, and carotenoids, thereby facilitating the formation of amylose inclusion complexes and enhancing the encapsulation of hydrophobic bioactives (12, 51). In addition, the relatively low steric hindrance and higher chain mobility of amylose favor molecular rearrangement and interaction with guest compounds, which further contributes to improved loading capacity and binding affinity in starch-based delivery systems. In contrast, amylopectin exhibits a highly branched architecture composed of short α-(1 → 4)-linked chains interconnected by α-(1 → 6) glycosidic bonds, resulting in a large, tree-like macromolecular structure. This extensive branching leads to a dense and spatially expanded network, which restricts chain mobility and reduces the accessibility of internal binding sites. As a consequence, amylopectin-rich systems generally show weaker inclusion capability toward small hydrophobic molecules but exhibit enhanced structural stability due to stronger intermolecular associations and entanglements. The branched structure also limits the ability of polymer chains to reorganize, thereby reducing the flexibility of the matrix and hindering the diffusion of encapsulated compounds.

Beyond these structural differences, the amylose/amylopectin ratio plays a decisive role in determining the encapsulation and release behavior of starch-based nanocarriers. An increased amylose proportion is typically associated with higher encapsulation efficiency due to its ability to form inclusion complexes and to interact with guest molecules through hydrophobic interactions (52). Amylose-rich systems under disordered or low-crystallinity conditions may also facilitate faster swelling and release, However, this behavior is strongly dependent on structural organization, as amylose crystallization and the formation of ordered domains can restrict water penetration and slow molecular diffusion. Meanwhile, higher amylopectin content generally promotes more entangled and compact networks, limiting diffusion pathways and contributing to more sustained release profiles (53).

The compositional effects produced by amylose and amylopectin are closely related to starch-based nanocarriers crystallinity, which ultimately determines supramolecular packing and mass transfer within the delivery system. Because crystalline arrangement differs across botanical sources, starches are classified into A, B, C, and V polymorphic types. A-type starches, commonly found in cereals, contain densely packed double helices with low water content. B-type starches, predominant in tubers such as potato, display looser helix packing and larger channel voids, enabling higher water retention. C-type starches are regarded as mixtures of A- and B-type arrangements. The V-type structure is distinct, arising only when amylose forms single-helix inclusion complexes with small hydrophobic guest molecules. Crystallinity refers to the proportion of ordered structural domains (e.g., double helices, crystalline lamellae) relative to amorphous regions within the granule (50, 54). Crystallinity in the here described starch nanostructures reflects reorganized or newly formed crystalline domains rather than native particle crystallinity. In this review, we focus on the crystallinity of starch nanocarriers fabricated through processes involving gelatinization, physical treatment, chemical hydrolysis, or enzymatic hydrolysis, which typically disrupt or transform the native crystalline domains. These modifications may lead to the dominance of amorphous structures, the formation of new crystalline organizations, or short-range ordering through recrystallization during cooling or retrogradation. Starch-based nanocariers with high crystallinity exhibit dense supramolecular packing that limits water penetration, swelling, and diffusion of encapsulated bioactives, thereby slowing or delaying release. Conversely, lower crystallinity or increased amorphousness produces a more open network, facilitating water ingress and accelerating cargo release. For example, one study showed that starch nanoparticles displayed markedly reduced relative crystallinity after nanoprecipitation, accompanied by enhanced water absorption but reduced oil-holding capacity (55). At the nanoscale, the disruption of crystalline order is prominent, meaning that nanostructured starch systems typically become more responsive and sensitive to release-triggering environments.

Starch crystallinity and amylose/amylopectin ratio could be interdependent and act synergistically to shape encapsulation and release behavior (27, 53). A starch matrix with a relatively high amylose content and moderately low crystallinity may offer both strong encapsulation capacity (via amylose helices) and rapid release (via its more accessible and hydrated amorphous domains) (12, 52). Conversely, when amylopectin content and crystallinity are both high, the resulting nanocarrier tends to form a rigid and compact network that stabilizes sensitive bioactives but restricts their diffusion, making such systems more suitable for delayed or sustained release purposes (54, 56).

In practical formulation, researchers modulate these parameters by selecting starch sources with different molecular architectures or applying pretreatments such as annealing, acid hydrolysis, or ultrasonication to tune crystalline structure (33–35, 55, 57, 58). This structural regulation is essential for balancing the dual objectives of delivery systems: protection of bioactives during processing and storage, and controlled release or enhanced absorption during digestion. A carrier that is overly crystalline may provide excellent protection yet hinder bioaccessibility, whereas an overly amorphous system may release cargo prematurely or become unstable in gastrointestinal conditions (27, 51). For instance, designing a starch nanocarrier intended for gastrointestinal sustained release may require an encapsulation system with higher crystallinity and higher amylose content, whereas rapid release applications may benefit from matrices richer in amylopectin and lower in crystallinity (52, 53). Overall, amylose/amylopectin ratio and crystallinity function as structural levers that dictate inclusion complex formation, molecular diffusion, hydration and swelling behavior, and ultimately the release kinetics and stability of bioactives within starch-based nanocarriers.

### 2.3 Composite and matrix effects

Beyond the use of native starch or its modified derivatives as a base (described in section 3), the design of composite and matrix materials represents a pivotal aspect in the effective delivery of nutraceuticals and bioactives. Composite and matrix materials refer to the introduction of additional nano-fillers, adjuvants, functional polymers, or structural modifications into the starch matrix to endow the system with improved structure, surface properties, release behavior, or targeting capabilities. These are generally categorized into two types:

1 Starch-polymer blends/interpenetration: Starch is blended with other natural or synthetic macromolecules (e.g., chitosan, cellulose, gelatin, proteins, or biodegradable polymers) to form starch-based complexes. This is done to intentionally alter hydrophilicity/hydrophobicity, mechanical and structural stability, and degradation rates.
2 Starch-based nanocomposites: Nanofillers (e.g., nanocellulose, nanoclays, carbon-based materials, metal oxide nanoparticles, etc.) are incorporated into the starch matrix to enhance the rigidity of the carrier structure, control porosity and wall thickness, and alter diffusion paths, thereby influencing release behavior (59, 60).

These composite/matrix systems play a crucial role in encapsulation and release performance. Encapsulation efficiency relies on the interactions between the matrix material and the bioactive substance, as well as between the bioactive and any fillers/adjuvants. Selecting an appropriate matrix or introducing functional fillers can enhance the affinity between the bioactive and the carrier, reducing initial release loss and thereby improving encapsulation efficiency. Release behavior depends on the structural stability of the carrier, the diffusion path of the bioactive, the degradation rate of the material, wall thickness, the barrier effect of fillers, and environmental responsiveness. In practice, researchers optimize the loading capacity, protective ability, release timing, and rate by adjusting the matrix composition. For example, to improve the encapsulation efficiency of hydrophobic bioactives, a modified starch-hydrophobic polymer blend may be selected (61, 62). To achieve intestinal targeted release, starch-based nanocarriers can be designed by introducing pH-sensitive functionalities or by increasing amylose content to form compact crystalline structures or V-type inclusion complexes (type of resistant starch), which stabilize the carrier under gastric conditions but allow enzymatic degradation and release in the intestine (12, 63, 64). Li et al. reported for example on chitosan hydrochloride-carboxymethyl starch nanogels that remain compact under gastric conditions due to suppressed ionization of carboxyl groups and strong covalent crosslinking, whereas ionization-induced electrostatic repulsion at intestinal pH leads to swelling and accelerated curcumin diffusion (65). Yu et al. found V-type cavities improved digestion resistance to upper-GIT digestion conditions while it enhanced colon targeting (66). Table 2 summarizes the performance of starch-based nanocomposites in bioactive delivery reported in recent years.

**Table 2:** Non-starch additives recently introduced into starch-based nanocarriers and their reported effects on encapsulation, release, and stability performance for nutraceutical delivery.

Non-starch additives	Effects on material properties	Encapsulated nutraceuticals	Encapsulation, release, and stability performance (changes after incorporation of non-starch additives)	References
Digestive enzyme coronas (α-amylase, pepsin, trypsin, lipase)	Protein layers altered surface charge, reduced particle size, and modified surface roughness	Quercetin	Thick corona→slow release; Thin corona→rapid release	(70)
Digestive enzymes coronas (pepsin, trypsin)		Quercetin		(147)
*Mesona chinensis* polysaccharide	Hydrogen bonding and steric effects inhibited particle aggregation, decreasing structural order	Curcumin	EE ↑; antioxidant activity ↑; GIT release time ↑	(114)
Dioscin (steroidal saponin)	Enhanced interfacial compatibility stabilized the interface, reducing aggregation tendency	Curcumin	EE and LC ↑; thermal/ionic/pH/ storage stability ↑; GIT release time ↑	(134)
Cinnamic acid	Aromatic conjugated systems enabled π–π and hydrogen bonding, enhancing carrier-bioactive affinity	Curcumin	Water solubility ↑; physical stability ↑; GIT release time ↑	(171)
Cinnamic acid		Curcumin	LC ↑; retention ↑; sustained GIT release ↑	(133)
Soy protein isolate	Protein incorporation introduced functional sites, increasing hydrogen-bonding and hydrophobic interactions	Curcumin	LC ↑; controlled and sustained release in SIF	(75)
Chitosan hydrochloride	Electrostatic and covalent crosslinking formed compact nanogel networks with high charge density and viscoelastic solid behavior	Curcumin	pH-responsive release with enhanced protection in SGF and accelerated diffusion in SIF	(65)
Choline chloride–lactic acid (CL)	Competitive hydrogen bonding disrupted crystalline structure, reducing particle size and starch-curcumin association	Curcumin	EE ↓; LC ↓	(124)
Sodium caseinate	Increased surface charge via complex formation, yielding more compact nanoparticles	Curcumin	EE ↑; LC ↑; intestinal transport efficiency and permeability ↑	(115)
Sodium caseinate	Electrostatic interaction with OSA starch formed a denser network structure and reduced particle size	Curcumin	EE ↑; LC ↑; faster release in SGF	(116)
Arabic gum	High water solubility and flexible polysaccharide chains promoted rapid matrix dissolution and efficient exposure of encapsulated substances	Chlorogenic acid	EE ↑; antiproliferative activity ↑; rapid release (≈100% within minutes)	(80)
Lecithin	Interfacial adsorption increased particle compactness and surface negative charge	β-lactoglobulin	Colon release time ↑; premature release in upper GIT ↓	(172)
Lecithin		Sinigrin	EE ↑; gastric stability ↑; controlled intestinal release	(160)
Sulfobetaine, deoxycholic	Formation of zwitterionic hydration layers (Sulfobetaine) suppressed protein adsorption and improved biocompatibility; introduction of hydrophobic domains (Deoxycholic) enabled self-assembly into micelles	Walnut peptide	Peptide loading ↑; stable nanomicelle formation	(151)
Citric acid, acetyl groups	Introduction of additional ester and carbonyl groups increased hydrogen-bonding sites and modified matrix polarity	Curcumin, ferulic acid	EE ↑	(56)
β-Cyclodextrin	Host–guest inclusion and hydrogen bonding provided hydrophobic cavities and steric protection, enhanced resistance to pepsin and acidic conditions, and promoted pH-responsive structural relaxation in alkaline media	Tea polyphenol	SGF release time ↓; SIF release time ↑ and complete colonic release; light/thermal stability ↑	(173)
Hollow mesoporous silica nanoparticles	High surface area and hollow mesopores increased loading sites; rigid nano-fillers reinforced network and delayed matrix rupture	Alliin	Diffusion and matrix erosion–controlled sustained intestinal release	(174)
Lysozyme	Electrostatic complexation with carboxymethyl starch and thermal gelation formed a 3D nanogel network, increasing thermal stability and structural integrity	Epigallocatechin gallate, anthocyanins	GIT release ↑; biocompatibility ↑	(123)
Trehalose	Formation of glassy matrix and hydrogen-bonding network reduced aggregation	Resveratrol	Redispersibility ↑; photostability ↑; maintained rapid dissolution	(81)
Biotin	Introduction of V_7_-type helical cavities and surface carboxyl groups increased host–guest interaction and surface charge	Resveratrol	LC ↑; Caco-2 uptake ↑; anti-inflammatory efficacy *in vivo* ↑	(148)
Zein	Hydrogen bonding, electrostatic and hydrophobic interactions with starch formed protective outer layer, and decreased surface hydrophobicity	Resveratrol	EE ↑	(76)
Zein		Curcumin	Gastric digestion stability ↑	(136)
Zein		Rutin	EE ↑; sustained release ↑; thermal/oxidative stability ↑	(175)
Zein		Rutin	EE ↑; thermal/pH stability ↑	(167)
Whey protein isolate		Rutin	EE ↑; antioxidant activity ↑	(166)
Fe^3+^	Coordination bonding formed pH-dependent networks and an amorphous surface layer; protonation at low pH weakened coordination	Tannic acid	>80% release within 30 min at pH 1.2–3.0; <50% release at pH 7.4 (60 min); ~90% retained at pH 9.0 (60 min)	(176)
Gum acacia	Incorporation of flexible polysaccharide chains provided hydrogen-bonding sites, improving network integrity and reducing brittleness	Kaempferol	loading stability ↑; sustained release ↑	(158)
Digestive enzymes (pepsin, trypsin, pancreatin containing amylase)	Electrostatic adsorption of enzymes on NH_2_-SNPs formed protein coronas (soft with pepsin; hard with trypsin) and amylase degraded starch matrix	Proanthocyanidin, epigallocatechin gallate, catechin, epicatechin	Sustained release; enzyme-responsive intestinal release	(66)
Gum arabic	Additional hydrogen-bonding and steric stabilization reduced aggregation and strengthened interfacial network	Delphinidin-3-O-sambubioside	EE ↑; thermal/pH/ionic stability ↑	(140)
Sodium alginate	Introduction of abundant carboxyl groups, increased negative surface charge, enhanced hydrogen bonding and electrostatic interactions with polyphenols	Apple polyphenols	Adsorption efficiency ↑; controlled release kinetics	(152)
Tween 80	Steric stabilization during nanoprecipitation reduced aggregation, enabling uniform SNP formation	Apple polyphenols	Enabled stable polyphenol–SNP complexes with effective adsorption	(154)
Casein	Protein network formation and electrostatic interactions with porous starch generated stable microgels with ordered secondary structure at pH 7.0	Phycocyanin	EE ↑	(122)
Bovine serum albumin	Protein–polyphenol and protein–starch interactions provide additional binding sites and steric stabilization, forming hybrid hydrogel nanoparticles	Polyphenolic compounds from *Moringa oleifera* leaf extract	Protection against oxidative/apoptotic degradation ↑	(177)

↑ indicates an increase or enhancement after adding non-starch additives, while ↓ indicates the opposite change, as described in the original studies. EE, encapsulation efficiency; LC, loading capacity; DS, degree of substitution; SGF, simulated gastric fluid; SIF, simulated intestinal fluid; GIT, gastrointestinal tract simulated fluids; OSA, octenyl succinic anhydride.

Native starch possesses functional hydroxyl groups and exhibits a structure where helical regions coexist within crystalline/amorphous domains. As a carrier base, starch is highly hydrophilic, swellable, and susceptible to water/enzyme ingress. When used as an encapsulation matrix without modification or fillers, it may lead to rapid diffusion and release in the gastrointestinal environment. Therefore, structural integrity (e.g., crystallinity, degree of crosslinking) must be considered to retard release. de Souza Falcão et al. (67) pointed out that when starch matrices encapsulate bioactive compounds, their crystalline vs. amorphous structure and hydration/swelling behavior are key influencing factors. For starch-polymer blend matrices, conversely, blending starch with other polymers can enhance encapsulation or controlled release by introducing additional hydrophilic/hydrophobic domains, crosslinked networks, or specific interactions at the carrier-bioactive interface. Furthermore, co-polymers can affect the rate of water or enzyme ingress, thereby delaying release. In starch-based nanocomposites, the addition of nanofillers to the starch matrix acts as a physical barrier layer to increase the diffusion path, slowing down the diffusion of bioactive molecules or enhancing matrix rigidity/degradation resistance. Fillers can also improve interactions with the matrix, thereby altering the localization of the bioactive substance or modifying water or enzymatic accessibility within the system.

In conclusion, researchers should consider the following at the material selection stage: the compatibility between the matrix and the bioactive substance; whether blends or fillers enhance stability or retard release; and the impact of the composite structure on bioactive localization, diffusion paths, and degradation response. Only in this way can the starch-based carrier be systematically optimized from the material design level to enhance the encapsulation, protection, and release performance of nutraceuticals.

## 3 Processing and preparation factors

The preparation and processing methods of starch-based nanocarriers dictate their microstructure, surface chemistry, and molecular organization. Consequently, they not only determine the initial formation of the carrier structure but also directly govern the entry, immobilization, and release pathways of bioactive substances. Understanding how these methods shape particle size, crystallinity, pore structure, and interactions is key to elucidating the encapsulation efficiency and release behavior of starch-based nanocarriers.

### 3.1 Preparation strategies and structural formation

There is a multitude of preparation methods for starch-based nanocarriers. The first category is top-down approaches, including ultrasonication, hydrothermal treatment, high-pressure homogenization, and ball milling. These methods employ mechanical shear, cavitation, and impact to fracture large starch granules into nanoparticles. The second category is bottom-up approaches, which involve dissolving or gelatinizing starch and then inducing the reorganization/regeneration of starch molecules into nanoparticles via a solvent/antisolvent system (nanoprecipitation). Emulsion methods involve forming water-in-oil or oil-in-water systems to shape starch or modified starch into nano-sized carriers within the emulsion droplets, followed by solidification into particles. While starch nanoparticles can also be used to stabilize Pickering emulsions, the starch matrix in these systems is not the primary determinant of bioactive encapsulation and starch nanoparticles as Pickering emulsifiers fall therefore outside of the scope of this review. As such, the emulsion-based approaches discussed here mainly focus on water-in-oil systems. Beyond physical preparation, chemical or enzymatic modifications (e.g., esterification, oxidation, debranching via pullulanase, hydrolysis, and crosslinking) are used to alter molecular structure, hydrophilicity/hydrophobicity, or gel network architecture (35, 68). Finally, post-processing techniques, such as spray drying or freeze-drying, are often employed to convert the carrier-bioactive complex into a final powder form (35, 69). These processes significantly impact the wall material structure and the carrier-bioactive interface.

### 3.2 Structure–function relationships governed by preparation methods

These preparation/processing methods are not merely isolated technical procedures; rather, they directly influence encapsulation capacity and release behavior by altering the key physicochemical properties of the nanocarrier. More importantly, different processing and preparation strategies determine the structural features of starch-based nanoparticles across multiple length scales, including particle size, surface morphology, internal organization (e.g., dense or porous matrices), and in some cases core–shell architectures, which collectively govern molecular interactions and mass transfer behavior within the carrier system (61, 67, 68). For example, physical reduction or precipitation methods typically yield smaller nanoparticles with increased specific surface area, thereby providing more binding sites or a larger contact interface for bioactive substances. At the nanoscale, size reduction is often accompanied by changes in surface morphology and roughness, which can enhance interfacial adsorption and improve accessibility of bioactives to the carrier matrix. Smaller particle sizes are generally advantageous for enhancing encapsulation efficiency, as bioactive molecules can more easily enter the carrier or adsorb onto the interface. However, smaller particles also imply shorter diffusion paths and greater interfacial exposure, which may lead to rapid release of the bioactive, thereby reducing the capacity for controlled release. Furthermore, disrupting crystallinity via physical methods enhances carrier accessibility for bioactives and encapsulation capacity but may compromise structural stability and accelerate release. Such structural disruption is also associated with the formation of more disordered internal regions, which facilitate solvent penetration, enzyme accessibility, and molecular mobility, thereby promoting faster release kinetics (66, 70). Precipitation, emulsion, or crosslinking methods can generate carriers with varying porosities, as well as hollow or core-shell structures (71–74). These architectures originate from differences in nucleation–growth processes, phase separation, and interfacial stabilization during fabrication, which determine internal density distribution and diffusion pathways (68). Carriers with high porosity and thin walls typically exhibit high loading capacity but faster release; conversely, structures reinforced by thick walls or crosslinking can retard release. In particular, core-shell structures enable spatial separation between a bioactive-rich core and a protective outer shell, thereby introducing an additional diffusion barrier and improving release controllability (75, 76). Surface chemical functionality also plays an important role in regulating bioactive loading and release. Accordingly, the use of adjuvants (e.g., surfactants, crosslinkers) in chemical/enzymatic modification or emulsion/solvent precipitation can alter the functional groups (–OH, –COOH, hydrophobic substituents) on the starch particle surface. This modifies the interaction between the starch matrix and the bioactive substance. For hydrophobic bioactives, hydrophobic modification can be achieved by introducing hydrophobic substituents (e.g., acetyl, alkyl, or fatty acyl groups) onto starch hydroxyl groups or by constructing hydrophobic cores in core–shell architectures, which enhances hydrophobic affinity, improves loading capacity and slows down release (15, 77, 78). Once the carrier is loaded with the bioactive, the transformation into a stable powder via spray drying, freeze-drying, or oven drying affects the compactness of the wall material, the localization of the bioactive, and the formation of cracks or pores within the structure, thereby influencing release behavior under storage, processing, and gastrointestinal conditions (79). Drying-induced structural rearrangement may further alter matrix density and pore distribution, which in turn affects permeability and release kinetics (69). Spray drying is an efficient encapsulation technique that enables the conversion of solutions, emulsions, and suspensions into solid particles in a single step, and has gained increasing attention in recent research. Nano-spray drying, in particular, allows the production of smaller particles compared to conventional spray drying, thereby enhancing the bioavailability of bioactive compounds and drugs. For example, Tobón-Vélez et al. encapsulated chlorogenic acid into starch–gum arabic carriers using nano-spray drying, significantly improving its inhibitory effect on colorectal cancer cells (80). Similarly, Xie et al. achieved a high loading capacity of 28.83% and an encapsulation efficiency of 91.28% for resveratrol using nano-spray drying, resulting in a 171.25% increase in relative bioavailability in rats, along with enhanced photostability and antioxidant activity (81). In another comparative study, nano-spray drying produced starch-based carriers with higher encapsulation efficiency for *Arthrospira platensis* extract than conventional spray drying and freeze-drying, while also yielding particles with improved color stability, thermal stability, and antioxidant capacity (69). However, it should be noted that the elevated temperatures involved in spray drying may lead to the degradation of heat-sensitive bioactives, and therefore the suitability of this technique should be carefully evaluated based on the thermal stability of the encapsulated compounds.

**Figure 1:**
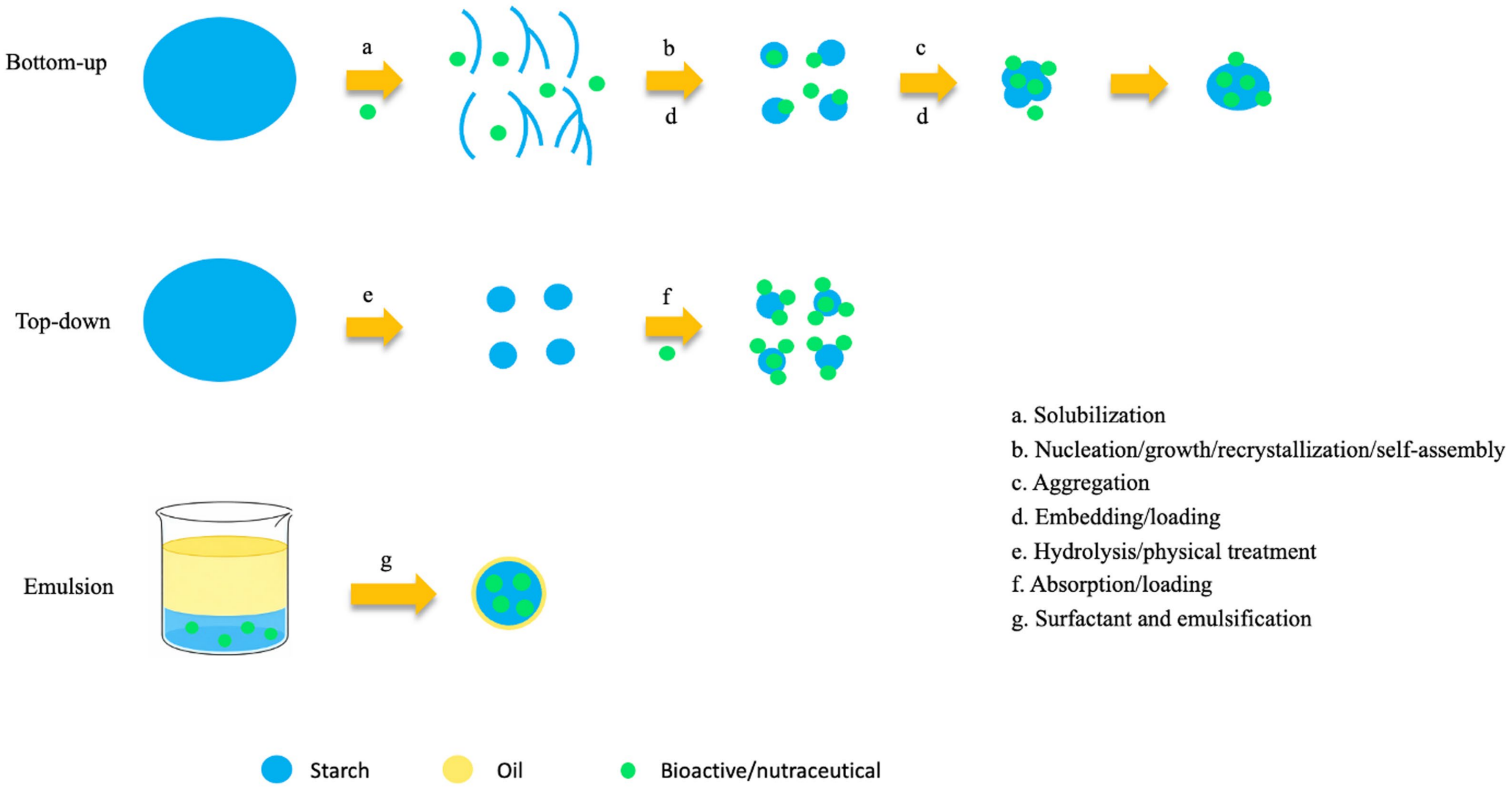
Bottom-up, top-down, and emulsion methods for preparing starch-based nanocarriers.

Table 3 summarizes the impact of preparation methods on carrier performance as reported in starch-based nanocarrier research in recent years. For delivery systems targeting nutraceuticals, high encapsulation efficiency and controlled release behavior are key performance indicators. Therefore, from a design perspective, understanding and selecting the appropriate preparation method is pivotal for optimizing carrier performance and enhancing the functional efficacy of bioactives. Figure 1 illustrates the preparation of starch-based nanocarriers via bottom-up, top-down, and emulsion approaches. Taking physical size reduction as an example, methods such as ultrasonication or ball milling reduce native starch granules to dimensions ranging from tens to hundreds of nanometers, enhancing contact with bioactive substances (82–86). This process also alters surface morphology and disrupts internal structure through mechanical forces and cavitation effects, which can increase surface energy and adsorption capacity. The mechanism involves particle size reduction, resulting in a larger specific surface area that promotes bioactive adsorption or encapsulation, while the disruption of crystalline structures, besides increasing the encapsulation potential, simultaneously also increases water and enzyme accessibility and accelerates release. As a result, these particles often show increased sensitivity to environmental conditions such as enzymatic digestion (66). Consequently, if the goal is rapid release, physical reduction is advantageous; however, if sustained release is desired, it may need to be combined with crosslinking or coating strategies. During antisolvent precipitation, starch is dissolved and subsequently added dropwise into ethanol, allowing precise control over particle size and size distribution. This method generates regular, tunable carriers favorable for uniform release. The precipitation conditions (e.g., solvent ratio, addition rate, and temperature) directly influence solvent–antisolvent mixing efficiency, nucleation rate, particle growth, and aggregation behavior, which collectively determine the colloidal properties of the resulting particles, thereby dictating the binding scenario between the bioactive and the carrier (35, 87–90). In terms of chemical/enzymatic modification and crosslinking, treatments such as acetylation, oxidation, or the addition of crosslinking agents serve to alter hydrophilicity, stabilize the structure, and slow down the rate of enzymatic hydrolysis (91–94). This enhances the retention of bioactive compounds within the carrier matrix, thereby mitigating initial burst release and improving sustained release capacity. Simultaneously, modification can improve the compatibility between the carrier and hydrophobic bioactives, leading to higher encapsulation efficiency.

**Table 3:** Recently reported preparation methods of starch-based nanocarriers and their effects on material properties, and related encapsulation, release, and stability performance for nutraceutical delivery.

Starch type	Encapsulated substances	Preparation methods	Effects on material properties	Encapsulation, release, and stability performance	References
Cassava starch	Anthocyanins	Enzymatic hydrolysis + nanoprecipitation	Enzymatic hydrolysis generated porous nanostarch with abundant binding sites; nanoprecipitation promoted rapid encapsulation	EE 78%; LC 76%; sustained release, and 52% retention after 21 days in GIT	(139)
Cassava starch	Berberine	Sulfuric-acid hydrolysis + nanoprecipitation	Acid hydrolysis generated small SNPs; nanoprecipitation promoted delivery system formation and stable dispersion	Controlled berberine migration in beef sausages; enhanced preservation performance	(178)
Cassava starch	Curcumin, ferulic acid	Nanoprecipitation + acetylation + citric-acid cross-linking	Nanoprecipitation generated amorphous SNPs; chemical modification altered polarity and cross-link density	EE 91%; diffusion-controlled release in food simulants	(56)
Waxy maize starch	Curcumin	Enzymatic debranching + gelatinization + retrogradation	Debranching and retrogradation generated loosely packed amylose-based matrices with accessible hydrogen-bonding domains, facilitating bioactive accommodation and diffusion	EE 63%; sustained release in GIT	(114)
Waxy maize starch	Curcumin	Enzymatic debranching + hydroxypropylation + self-assembly	Debranching generated short amylose chains; hydroxypropyl groups increased hydrophilicity; self-assembly produced compact nanospheres	Water solubility increased 226-fold; physical stability increased 5.3-fold; sustained release in GIT	(132)
Waxy maize starch	Curcumin	Acid-ethanol hydrolysis + cinnamic acid esterification + emulsion solvent method	Hydrolysis reduced starch chain length enabling nanoparticle formation; esterification introduced amphiphilicity and aromatic sites	LC 27%; sustained release in GIT	(171)
OSA starch	Curcumin	pH-driven method + high-pressure dynamic microfluidization	pH-driven method increased surface charge	EE 89%; LC 24%; improved transport efficiency and permeability	(115)
Tapioca starch	Astaxanthin	High-speed jet processing	High-speed jet generated small, low-crystallinity starch nanocarriers	Sustained *in vivo* efficacy; *C. elegans* lifespan increased by 22–31%	(179)
	Chlorogenic acid	Nano spray-drying	Formation of spherical nanoparticles with smooth surfaces and homogeneous distribution	EE 101–113%; rapid release in PBS	(80)
Debranched potato starch	β-lactoglobulin	Nanoprecipitation + OSA modification + self-assembly	OSA modification introduced amphiphilicity	Sustained colon release; reduced upper-GIT loss	(172)
Maize starch	Saturated fatty acids (C4–C18)	Solid-state acid hydrolysis + citric acid esterification + nanoprecipitation	Citric acid esterification increased hydrophobicity; nanoprecipitation promoted spherical composite formation	Higher citric acid-modified starch nanoparticles content slowed release	(180)
High amylose corn starch, potato starch	Vitamin D_3_	Ultrasonication + low-temperature processing	Ultrasonication disrupted crystalline regions, reduced particle size, generated uniform starch nanoparticles with high surface area and adsorption sites	EE 95%; EL increased with sonication time; improved colloidal stability	(52)
Corn starch (native, high amylose)	Vitamin D_3_	Nanoprecipitation + self-assembly + ultrasonication		LE > 90%; sustained, diffusion-controlled release under pH 7.4, 37 °C	(150)
Maize starch	β-carotene	Dry heating + self-assembly	Controlled cross-link density; tunable particle fragmentation and hydrophobicity	Enhanced inner loading and improved thermal stability	(155)
Sorghum starch, foxtail millet starch, pearl millet starch	β-carotene	Acid hydrolysis + ultrasonication	Acid hydrolysis reduced granule size and generated nanoporous structure; ultrasonication promoted uniform dispersion and encapsulation	EE 90%; sustained release in GIT with higher intestinal bioaccessibility	(58)
Corn starch	Walnut peptide	Chemical grafting + self-assembly	Amphiphilic starch self-assembled into uniform spherical nanomicelles with smooth surface	EE > 90%; pH-responsive controlled release	(151)
Corn starch	Alliin	Hollow mesoporous silica nanoparticles adsorption + Ca^2+^-crosslinked	Dual encapsulation generated hierarchical barrier, enhancing mechanical integrity and pH sensitivity	Targeted and sustained release in SIF over 36 h	(174)
Carboxymethyl starch	Epigallocatechin gallate, anthocyanins	Electrostatic self-assembly + self-assembly thermal gelation	Heating-induced protein denaturation and network formation yielded uniform nanogels with high stability	EE 80%; pH-responsive, sustained release in GIT	(123)
Cassava starch	Quercetin	Acid hydrolysis + gelatinization + graft copolymerization	Acid hydrolysis generated crystalline nanocrystals with porous/rough morphology and high surface hydroxyl density	EE 81%; sustained release 84% at 24 h in PBS	(181)
Debranched waxy maize starch	Quercetin	Enzymatic debranching + nanoprecipitation + surface oxidation or amination	Debranching generated short-chain starch; nanoprecipitation produced nanoscale particles with tunable surface charge	EE 75%; sustained release in GIT	(147)
Debranched waxy maize starch	Sinigrin	Enzymatic debranching + self-assembly	Self-assembly generated monodisperse SNPs with enhanced binding sites for hydrophilic molecules	EE 69%; sustained release in SIF; high resistance to acidic degradation	(160)
Indica rice starch	Capsaicin	Enzymatic debranching + recrystallization	Debranching generated short-chain amylose and recrystallized nanoparticles with porous matrix and abundant binding sites	EE 70%; LC 13%; sustained release in 50% ethanol/PBS/GIT	(182)
Arrowhead starch, bracken starch, kudzu starch	Lactoferrin	Ball milling + alkaline pretreatment + homogenization + ultrasonication	Ball milling reduced granule size and increased surface area; homogenization and ultrasonication facilitated uniform nanoparticle formation	EE 75–84%; sustained protection in SGF with release in SIF	(156)
Waxy maize starch	Proanthocyanidin, epigallocatechin gallate, catechin, epicatechin	Nanoprecipitation + enzymatic debranching + chemical amination	Amination introduced positive charges and enhanced electrostatic interaction with polyphenols	Enzyme-responsive intestinal release	(66)
Oxidized potato starch	Anthocyanins	Reverse-emulsion + sodium trimetaphosphate crosslinking	Formation of uniform, spherical, solid starch nanocarriers with high structural stability	Sustained release in GIT	(183)
Lotus stem resistant starch	Kaempferol, crocin	Planetary ball milling + ultrasonication	Ball milling generated nano-sized particles with higher surface area and negative charge; ultrasonication improved dispersion and loading uniformity	EE 62–76% (crocin), 68–81% (kaempferol); increased post-extrusion retention	(159)
Waxy corn starch	*Eruca sativa* polyphenols	Ultrasonication + nanoprecipitation	Ultrasonication produced nanoscale starch and amorphous structure; nanoprecipitation enabled polyphenol incorporation into nanocapsules	EE 16%	(161)
OSA starch	Curcumin	pH-driven method + high-pressure dynamic microfluidization	Generated smaller nanoparticles with network-like morphology	EE 89%; LC 24%; sustained release during *in vitro* digestion	(116)
Horse and water chestnut starch; lotus stem starch	Resveratrol	Ball milling + ultrasonication	Formation of porous/film-like starch nanoparticles with internal channels for physical entrapment	EE 76–81%; sustained release in SGF; fast release in SIF	(184)
Sago starch	Piperine	Chemical acetylation + nanoprecipitation	Acetylation generated hydrophobic starch derivatives; nanoprecipitation produced uniform spherical nanoparticles	LC 0.50 mg/mg; and sustained release in GIT	(164)
Hydroxyethyl starch	Curcumin	Esterification + nanoprecipitation and dialysis	Formation of amphiphilic conjugates enabled micellar self-assembly with low critical micelle concentration and stable nanostructure	LE 13%; sustained and acid-responsive release	(135)
Corn starch	Apple polyphenols	Ultrasonication + nanoprecipitation	Ultrasonication and ethanol disrupted crystalline regions and increased surface –OH availability	AC 2.65 μg/mg; improved antioxidant stability; sustained release in GIT	(154)
Corn starch	Apple polyphenols	Acid/enzymatic treatment	Generated nanoscale starch with increased surface area and accessible adsorption sites	AC 81.0 mg/g; sustained release in GIT	(153)
Tapioca starch	Myricetin	High-speed jet treatment	Generated uniform nanostarch with high surface area and accessible binding sites	LC 103.13 mg/g; sustained release in GIT; improved antioxidant stability	(165)
*Agriophyllum squarrosum* starch	Lycopene	Ionic-liquid-assisted dissolution + ultrasonication + nanoprecipitation	Generated small nanoparticles with effective inclusion sites and improved environmental stability	High retention under pH/temperature/light; sustained release during *in vitro* digestion	(168)
Corn starch	Curcumin, D-*δ*-tocopherol, Coenzyme Q10	Coprecipitation near melting point of guest + spray-drying/freeze-drying	Heating near guest melting point induced spherical aggregation with SNPs via hydrogen bonding and hydrophobic interactions; drying method tuned porosity and density	Freeze-dried composites showed higher stability for curcumin and D-δ-tocopherol; spray-dried composites showed higher stability for Coenzyme Q10	(185)
Waxy maize starch	Resveratrol	Wet milling + spray drying	Wet milling reduced particle size and crystallinity; spray drying formed spherical microcapsules with smooth surfaces and good redispersibility	LC 29%; EE 91%; rapid dissolution; bioavailability 171%	(81)
Debranched maize starch	Tangeretin	Ultrasonication + recrystallization	Ultrasonication promoted molecular collision and ordered recrystallization; longer sonication increased nanostructure crystallinity	RI 99% after 20 days	(121)
Glutinous rice starch	Phycocyanin	Ultra-high-pressure homogenization	Homogenization promoted molecular ordering of casein and intimate integration with porous starch, forming uniform polygonal microgels	EE 98%; LC 43%; sustained release over 48 h in PBS	(122)
Debranched waxy maize starch	Epigallocatechin gallate	Ultrasonication + recrystallization	Generated uniform nanoparticles with reduced PDI and B-type crystallinity	EE 88%; LC 23%; improved colloidal stability at neutral pH	(186)
Debranched waxy maize starch	Tea polyphenols	Vacuum oxygen cold plasma	Surface oxidation generated carboxyl groups and increased surface area	Higher AC and loading with longer cold plasma treatment time	(170)
Corn starch	Polyphenolic compounds from *Moringa oleifera* leaf extract	Alkaline gelatinization + dialysis	Formation of spherical nanoparticles with stable surface charge	Sustained bioactivity and improved *in vivo* hepatoprotective performance	(177)
Debranched waxy corn starch	Curcumin	Acid-ethanol hydrolysis/enzymatic debranching + acetylation + nanoprecipitation	Pretreatment reduced molecular weight; acetylation enabled nanoparticle self-assembly	LE 55%; LC 3%	(57)

EE, encapsulation efficiency; LC, loading capacity; LE, loading efficiency; EL, encapsulation loading; AC, Adsorption capacity; RI, retention index; OSA, octenyl succinic anhydride; SGF, simulated gastric fluid; SIF, simulated intestinal fluid; GIT, gastrointestinal tract simulated fluids; PBS, Phosphate-buffered saline.

In summary, by selecting appropriate preparation and processing methods, and by considering particle size control, structural compactness, degree of modification, carrier-bioactive interactions, and final powder morphology, researchers can systematically regulate the encapsulation and release behavior of starch-based nanocarriers. For most factors discussed in this review, preparation/processing methods are, in essence, the means to control these factors. When designing high-efficiency nutraceutical carriers, it is imperative to consider not only the carrier material itself but also how the processing route shapes its final functional characteristics.

## 4 Active compound factors

When evaluating the encapsulation and release behavior of starch-based nanocarriers for nutraceuticals, the intrinsic properties of the active compound itself often serve as the primary determinants dictating system performance. The ability of starch materials to effectively encapsulate, provide stable protection for, and release target molecules on demand hinges on specific characteristics, including the solubility, molecular size, charge, chemical stability, and intermolecular interaction potential of these bioactives. Only by elucidating these intrinsic driving forces from the perspective of the active compound can one explain why certain bioactives are efficiently encapsulated while others rapidly escape. Furthermore, this understanding is essential to comprehend why distinct bioactives exhibit vastly different release kinetics within an identical starch carrier system. Consequently, this section focuses on active compound factors, analyzing the specific physicochemical mechanisms through which they influence encapsulation efficiency, structural stability, and medium-triggered release behaviors. Combined with relevant research examples, this discussion highlights the fundamental role these factors play in enhancing the effective delivery of nutraceuticals.

### 4.1 Hydrophilicity and hydrophobicity

Hydrophilicity and hydrophobicity are critical properties describing the partitioning tendency of bioactive molecules between polar and non-polar media, and, as such, they are also core determinants governing how these molecules are incorporated into the interior of starch-based nanostructures during preparation. Bioactive substances with high water solubility tend to remain in the aqueous phase environment and are prone to escaping from the particles during carrier formation, typically resulting in lower encapsulation efficiency (95–100). Simultaneously, when such molecules exist within the carrier structure solely through physical encapsulation, diffusion resistance is minimal, facilitating rapid release during storage and application. Therefore, in practical operations, techniques such as crosslinking, precipitation, or coating are commonly employed with starch materials to reduce the diffusion and escape of these bioactives. In contrast, hydrophobic bioactives exhibit distinctly different behavior. These molecules readily engage in hydrophobic interactions favorable for stable binding within hydrophobic microdomains formed after starch modification, inside the helical cavities of amylose, or within cyclodextrins. Consequently, encapsulation efficiency tends to be higher, as bioactive molecules are spatially confined and exhibit reduced mobility within the carrier matrix (101, 102). During the release phase, hydrophobic bioactives diffuse more slowly within the carrier, and the release rate is often dominated by a “diffusion-controlled” mechanism. Accordingly, when sustained release is desired for hydrophobic bioactives, carrier design strategies that strengthen hydrophobic encapsulation domains are generally preferred.

### 4.2 Molecular weight and size

Molecular weight and molecular dimensions are key factors influencing intra-particle diffusion kinetics and the spatial accommodation of bioactive substances within carriers (103, 104). Large-sized or high-molecular-weight bioactives necessitate sufficient pore size or nanoscale voids to penetrate the particle interior. This implies that the carrier must retain voluminous cavity structures or a looser network architecture during the preparation process (105). If porosity is inadequate, such molecules tend to reside on the particle surface, leading to increased surface loading and compromised encapsulation stability (106). Once encapsulated, macromolecules exhibit low diffusion rates and slow desorption due to their large size and limited mobility, as a result, they tend to show sustained release behavior (107). Conversely, low-molecular-weight bioactives readily penetrate the particle interior but are also more prone to diffusion and migration within the external medium, which results in a higher susceptibility to issues such as rapid release, inadequate protection, or premature leakage (108, 109). This characteristic can be viewed as either an advantage (facilitating rapid release) or a disadvantage (causing uncontrolled release or premature degradation). Therefore, in carrier design, it is often necessary to tailor parameters such as the pore size, degree of crosslinking, nanoparticle dimensions, and shell thickness of the starch carrier according to the specific size of the bioactive substance.

### 4.3 Intermolecular interaction capability

Intermolecular interaction capability refers to the ability of bioactive substances to associate with starch constructs at the molecular level via mechanisms such as hydrogen bonding and hydrophobic effects. This property determines the binding strength within the carrier, the localization of embedment, and the contribution to particle stability. The hydroxyl groups of bioactive substances readily bind to the abundant hydrogen-bonding sites on starch molecules, thereby enhancing affinity during encapsulation. Furthermore, bioactive substances capable of forming stable inclusion complexes, such as hydrophobic guests within amylose or cyclodextrins, are more amenable to forming structurally stable encapsulations following nanonization. These specific binding interactions can significantly improve encapsulation efficiency and reduce release rates. Conversely, if the bioactive substance exhibits negligible specific binding with starch, the system must rely solely on physical entrapment mechanisms, where release behavior is predominantly governed by diffusion. Specifically, charge describes the electrical characteristics of the bioactive substance in a given medium, while polarity reflects the distribution features of its electron cloud. These two properties directly determine whether electrostatic attraction, hydrogen bonding, or dipole interactions can form between the bioactive and the starch molecules or chemically modified carrier materials (110). For instance, anionic phenolic compounds can form strong electrostatic associations with cationized starch nanoparticles, thereby significantly enhancing encapsulation stability (111–113). It is important to note that charged or highly polar molecules are typically readily soluble in water, leading to rapid diffusion into the aqueous phase during preparation and rendering effective encapsulation more challenging. Therefore, during the preparation process, it is often necessary to adjust the pH, introduce oppositely charged ligands, or utilize intermolecular hydrogen bonding to strengthen the interactions, thereby preventing the premature desorption of the bioactive substance during preparation or storage.

To explicitly connect nutraceutical properties with carrier design, it is essential to consider how bioactive characteristics interact simultaneously with carrier material, preparation methods, and other governing factors discussed in this review. The physicochemical properties of nutraceuticals, including solubility, molecular size, charge, and chemical stability, not only determine their intrinsic encapsulation tendency but also dictate how structural and colloidal features should be designed. For instance, hydrophilic bioactives, which exhibit high aqueous solubility and diffusion rates, are more prone to leakage during carrier formation. Therefore, their effective encapsulation requires carriers with higher matrix compactness and stronger intermolecular interactions, which can be achieved through increased crystallinity, composite reinforcement (e.g., polysaccharide or protein incorporation), as well as processing strategies that influence structural organization and water accessibility (55, 114–116). In addition, colloidal properties such as particle size and aggregation behavior must be carefully controlled to minimize surface-associated loss and improve retention. In contrast, hydrophobic bioactives benefit from enhanced affinity with hydrophobic domains, such as modified starch matrices or amylose-based inclusion structures. In these systems, molecular interactions are considered to play a dominant role in stabilizing the encapsulated compound and controlling release behavior, and can be further tuned through chemical modification, composite effects, or carrier type selection, including core–shell structures and inclusion complexes (15, 77, 78). The effectiveness of these strategies also depends on preparation methods, as processes such as nanoprecipitation or self-assembly determine how hydrophobic domains are formed and how the bioactive is spatially distributed within the carrier.

Table 4 summarizes the influence of active compound properties on the encapsulation and release behaviors of starch-based nanocarriers. In conclusion, when designing an effective starch-based nanocarrier system for nutraceutical delivery, it is advisable to first systematically evaluate the physicochemical characteristics of the active molecule. Subsequently, the carrier should be tailored based on these factors, for instance, by selecting appropriate starch sources and nanostructures, determining the necessity of chemical modification, adjusting nanoparticle size, regulating the hydrophilic–hydrophobic balance, and engineering specific release trigger mechanisms. Only through such an integrated and interaction-driven design approach can improvements in encapsulation efficiency, release profiles, and bioavailability be substantially realized.

**Table 4:** Recently reported nutraceuticals encapsulated by starch-based nanocarriers, their physicochemical properties and effects, and encapsulation, release, and stability performance.

Encapsulated nutraceuticals	Physicochemical properties	Effects	Encapsulation, release, and stability performance	References
Anthocyanins	Hydrophilic; small molecules; multiple phenolic –OH; pH-dependent charge	H-bonding, hydrophobic and electrostatic interactions drive adsorption onto SNPs	EE 78%; LC 76%; sustained release in GIT	(139)
			Enhanced thermal stability (>150 °C)	(53)
			Improved oxidative damage protection; higher tissue retention	(142)
Curcumin	Hydrophobic; H-bonding and π–π interaction capability	H-bonding and hydrophobic interactions with starch	Low SGF release (4%) and controlled SIF release (39%); 2-fold increase in oral bioavailability	(183)
		Hydrophobic, π–π and H-bond interactions with starch	LE 55%; LC 3%; improved colloidal stability in aqueous system	(57)
			EE 89%; LC 24%; first-order release; permeability increased 2-fold	(115)
			EE 63% and sustained release in GIT	(114)
			Sustained release in GIT and significantly enhanced bioavailability	(134)
			EE 86%; photodegradation reduced; sustained release	(131)
			Enhanced solubility (18-fold); sustained release in GIT; improved physical stability (6–8-fold)	(171)
			EE 81–91%; sustained release in food simulants	(56)
			EE 87%; sustained release in GIT	(75)
			Increased water solubility (226-fold); Increased physical stability (5.3-fold); superior *in vitro* anti-inflammatory activity; sustained release in GIT	(132)
			Increased water solubility (92-fold); enhanced dissolution and PAMPA permeability; higher cell uptake; improved thermal and light stability	(137)
			EE 89%; accelerated release in SGF; sustained release in SIF	(116)
			LE 66%; LC 21.64 mg/g; improved UV /thermal stability; sustained release in GIT	(138)
		Hydrophobic interactions with zein core and H-bonding with starch	EE 97%; sustained release in GIT; enhanced antioxidant activity	(136)
Astaxanthin	Hydrophobic; large, conjugated molecule; hydrophobic and H-bonding capability	Promoted embedding into amorphous starch-based nanostructure regions and suppressed recrystallization	Sustained functional efficacy; reduced intestinal lipofuscin by 42% after 5 days	(179)
Berberine	Hydrophobic; aromatic planar structure; H-bonding and π–π interaction capability	Hydrophobic, π–π and H-bonding interactions promoted nano-complex formation	Sustained release; effective lipid/protein oxidation suppression in beef sausages	(178)
Chlorogenic acid	Hydrophilic; multiple phenolic –OH; low membrane permeability	Strong affinity for hydrophilic polysaccharide matrices; rapid diffusion from water-soluble carriers	EE > 100%; fast release; enhanced cytostatic activity	(80)
Resveratrol	Hydrophobic; low molecular weight; multiple phenolic –OH	Hydrophobic interactions and low molecular weight promoted compact assembly	EE 91%; accelerated dissolution; improved photostability; enhanced bioavailability	(81)
Resveratrol			LC 20%; improved colonic efficacy of resveratrol, alleviating mice colon shortening (~25% recovery)	(148)
β-lactoglobulin	Protein bioactive	Strong interaction with starch–lecithin matrix formed compact and digestion-resistant structure	Colon-targeted release; GLP-1 secretion increased by 169% after fermentation; stable during upper-GIT transit	(172)
Saturated fatty acids (C4–C18)	Hydrophobicity increases with chain length; low chemical activity	Higher hydrophobicity promoted surface localization and increased composite hydrophobicity, acting as a diffusion barrier	Delayed GIT release for longer-chain fatty acids; improved storage stability	(180)
Procyanidins (PAC); epicatechin (EC); catechins (C)	Hydrophilic; multiple phenolic –OH; molecular weight (C/EC < PAC)	Larger molecular weight promoted stronger adsorption via multipoint interactions	PAC showed highest adsorption amount	(187)
Vitamin D_3_	Hydrophobic; H-bonding capability	Hydrophobic interactions, H- bonding and van der Waals forces with amylose-rich nanodomains	EE 95%	(52)
			Improved stability of vitamin D₃ in fortified milk and slower release in GIT	(149)
			High loading (>90%); improved stability; diffusion-controlled sustained release	(150)
Catechin (C), epicatechin (EC), epigallocatechin gallate (EGCG), Procyanidins (PAC)	Hydrophilic; multiple phenolic –OH; molecular weight C/EC < EGCG < PAC	Higher molecular weight and additional functional groups increased binding sites and intermolecular H-bonding with starch	Increased loading amount with molecular weight; PAC showed highest LE	(143)
Catechin, epicatechin, epigallocatechin gallate, procyanidins	Hydrophilic; multiple phenolic –OH; negatively charged in aqueous media	Electrostatic attraction and H-bonding with NH_2_-SNPs	Sustained release in GIT	(66)
Epigallocatechin gallate	Hydrophilic; multiple phenolic –OH	Multipoint H-bonding and electrostatic interactions with starch nanogel	EE 80%; sustained release and improved stability in GIT	(123)
			EE 80–88%; LC 21–23%; enhanced antioxidant and antibacterial activity; improved stability	(186)
β-Carotene	Hydrophobic	Hydrophobic interaction with starch	Enhanced inner loading and improved thermal stability	(155)
			Improved antioxidant activity; sustained release in GIT	(58)
Walnut peptide	Hydrophilic; low molecular weight	Compatible with hydrophilic micellar core	Sustained release in GIT; 41% absorption in duodenum at 120 min; enhanced pH/thermal/ hemolytic stability	(151)
Tea polyphenol	Amphiphilic, multiple phenolic –OH	H-bonding and hydrophobic interactions with starch	EE 87%; sustained release in GIT; complete release in colon; improved bioavailability and functional stability	(173)
Alliin	Hydrophilic; small molecular weight; multiple polar groups	H-bonding interaction with starch	Sustained release in GIT; retained antioxidant activity	(174)
Quercetin	Hydrophobic; multiple phenolic –OH	H-bonding and hydrophobic interactions with starch	Sustained release; enhanced antitumor efficacy *in vitro* and *in vivo*; improved survival rate in mice	(146)
			Sustained release	(147)
			EE 85%; enhanced stability; increased bioaccessibility	(188)
			EE 81%; sustained release reaching 84% at 24 h	(181)
Lactoferrin	Protein with multiple functional domains	Encapsulation restricted molecular mobility and shielded protein from enzymatic degradation	Retained inhibition of α-amylase, α-glucosidase, and DPP-IV after *in vitro* digestion; improved antidiabetic activity	(156)
Chrysin, rutin	Hydrophobic; multiple phenolic –OH	H-bonding and hydrophobic interactions with SNPs	EE 73–79%; sustained release in GIT; enhanced antioxidant activity; storage retention >90% (chrysin) and >85% (rutin) after 15 days	(157)
Tannic acid	Hydrophilic; multiple phenolic –OH; pH-responsive coordination with Fe^3+^	Multipoint coordination and H-bonding create compact interfacial network; acidic pH triggers disassembly	Strong antioxidant and antibacterial activity at pH 1.2–3.0; minimal activity and release at pH 7.4–9.0	(124)
Kaempferol	Hydrophobic; low molecular weight; limited –OH	Low polarity and small molecular size favor hydrophobic association with SNPs	Improved protection and sustained release	(158)
Crocin	Hydrophilic; high molecular weight; multiple –OH and glycosidic moieties	Multiple –OH and glycosidic structure generate strong polarity and localized charge distribution in aqueous media, promoting strong association with negatively charged SNPs surfaces and formation of compact matrices	High retention and improved thermal stability	(159)
Propolis polyphenols	Multiple phenolic –OH	H-bonding interaction with starch	Improved antioxidant retention; degradation <30% at 115 °C; enhanced shelf-life prediction under isothermal modeling	(130)
Delphinidin-3-O-sambubioside	Hydrophilic; multiple –OH	H-bonding interaction with dextrin	EE 93%; sustained release in GIT; 90% retention	(140)
Sinigrin	Hydrophilic; low molecular weight; multiple polar functional groups	High polarity and small molecular size favored accommodation within amorphous SNPs domains	Limited SGF release and sustained SIF release	(160)
*Eruca sativa* polyphenols	Hydrophobic; multiple phenolic –OH	Hydrophobic association with succinic groups; H-bonding with starch chains	EE 16%	(161)
Luteolin	Hydrophobic; multiple phenolic –OH	Hydrophobicity and multipoint H-bonding favor embedding into amorphous oxidized SNPs domains	EE 87%; limited SGF release and sustained SIF release; enhanced antioxidant stability	(163)
Piperine	Hydrophobic	Hydrophobic interactions between piperine and starch acetate matrix	LC 0.50 mg/mg; sustained release; improved retention	(164)
Apple polyphenols	Hydrophilic; multiple phenolic –OH; partially ionizable	H-bonding and electrostatic interactions with alginate-modified starch	Improved storage and GIT stability	(153, 154)
	Hydrophilic; multiple phenolic –OH	H-bonding interaction and adsorption onto SNPs	Improved antioxidant stability; limited SGF release and sustained SIF release	(152)
Phenolic compounds from green propoli	Hydrophobic; multiple phenolic –OH; pH-dependent ionization	Increased hydrophobicity and surface charge of SNPs	LE 65–73%; sustained release in GIT release; improved stability during digestion	(189)
Myricetin	Hydrophobic; multiple phenolic –OH	Low solubility favors surface adsorption; H-bonding with starch chains	AC 103.13 mg/g; sustained release in GIT; improved antioxidant stability	(189)
Rutin	Hydrophobic; multiple phenolic –OH	H-bonding and hydrophobic interactions with starch	EE 86%; sustained release; enhanced thermal/oxidative stability	(175)
			Increased DPPH scavenging and ABTS scavenging; sustained release in GIT	(166)
			EE 74%; enhanced thermal/pH stability	(167)
Lycopene	Hydrophobic	Hydrophobic interactions with SNPs	EE 64%; improved light/thermal/pH stability; digestive retention 77%; sustained release in GIT	(168)
D-δ-tocopherol	Hydrophobic; low melting point	Melted state during processing favored intimate association with SNPs	Enhanced stability and increased retention	(185)
Coenzyme Q10	Hydrophobic; bulky quinone structure; high molecular weight	Strong hydrophobic character and bulky molecular architecture favored compact co-aggregation with starch nanoparticles during coprecipitation	Enhanced pH/thermal/UV stability	(185)
Tangeretin	Hydrophobic	Hydrophobic interactions with cyclodextrin	High stability during storage (RI 93–99% at 20 days); effective protection against deterioration	(186)
Phenolic compounds from propolis	Hydrophobic; multiple phenolic –OH	H-bonding and hydrophobic interactions with starch	LE 55–71%; improved storage stability	(169)
Phycocyanin	Hydrophilic; macromolecule; charged amino acid residues	Hydrophilicity, macromolecular nature, and electrostatic interaction improve retention within porous starch–casein network	EE 98% and LC 43%; sustained release; preserved fluorescence	(122)
Tea polyphenols	Hydrophilic; multiple phenolic –OH; pH-dependent ionization	Hydrophilicity favors aqueous dispersion; multipoint H-bonding and electrostatic attraction promote adsorption onto negatively charged SNPs surfaces	LC 47.26 μg/mg; improved dispersion stability	(170)
Phenolic compounds from *Moringa oleifera* leaf extract	Hydrophilic; multiple phenolic –OH	Multipoint H-bonding interaction with starch/protein matrix	Improved protection and *in vivo* functional efficacy	(177)

SNPs, starch nanoparticles; EE, encapsulation efficiency; LC, loading capacity; LE, loading efficiency; AC, absorption capacity; RI, retention index; CaSR, calcium-sensing receptor; GLP-1, glucagon-like peptide-1; SGF, simulated gastric fluid; SIF, simulated intestinal fluid; GIT, gastrointestinal tract simulated fluids; PAMPA, parallel artificial membrane permeability assay.

## 5 Types of starch-based nanocarriers

**Figure 2:**
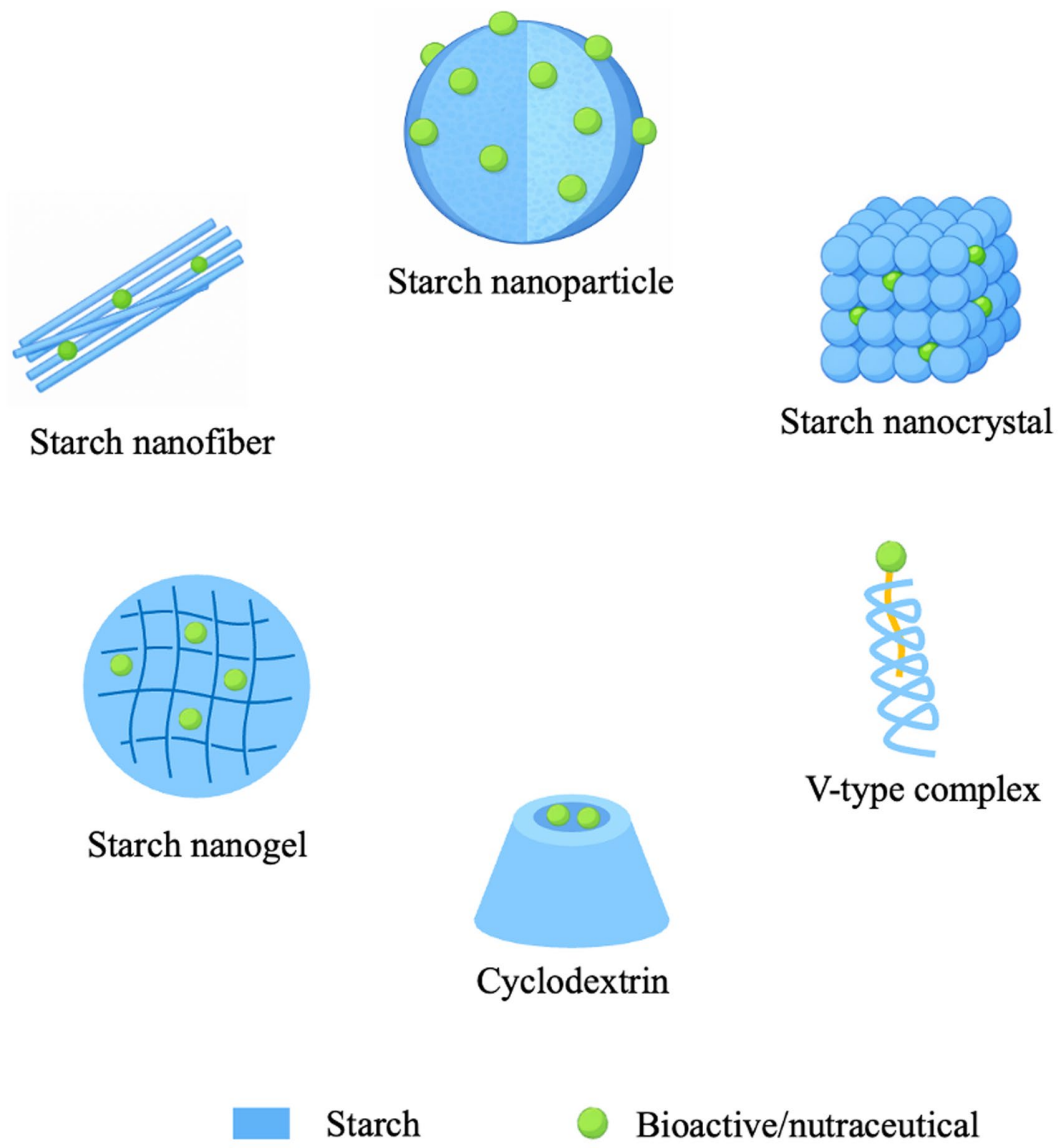
Common starch-based nanostructures used for nutraceutical delivery.

Having systematically discussed the intrinsic material (i.e., starch) properties, processing and preparation parameters, as well as the intrinsic properties of the to-be-encapsulated bioactive compounds, it is necessary to return to the structural types of the carrier system itself. Although different starch-based nanocarriers share a common foundation of natural starch macromolecules, they exhibit significant distinctions in morphology, crystallinity, interfacial characteristics, spatial conformation, and internal diffusion paths. These structural variations fundamentally dictate their interaction modes with bioactive substances and their ultimate delivery performance. Current research has developed a variety of starch-based nanostructures applicable to food and nutraceutical systems, including nanoparticles, nanocrystals, nanofibers, nanogels, V-type helical inclusion complexes, and cyclodextrin derivatives. Figure 2 illustrates the commonly reported starch-based nanostructures employed for nutraceutical delivery, highlighting their representative morphologies and organizational features. Understanding the structural logic and performance disparities among these types not only complements the framework of influencing factors discussed previously but also provides a clear structural basis for selecting the appropriate delivery platform.

### 5.1 Starch nanoparticles

Starch nanoparticles (SNPs) refer to particles formed by dispersing or exfoliating starch (or its modified forms) to nanoscale dimensions (<1,000 nm) via physical, chemical, or enzymatic methods (15, 42, 51, 68). They are the most common carriers in delivery systems, beneficial for adsorbing bioactive substances and enhancing carrier-bioactive interactions. Due to their small size, high specific surface area, and surface rich in hydroxyl groups (derived from the glucose units of starch), SNPs can bind with active molecules via physical adsorption, hydrogen bonding, and Van der Waals forces. As summarized previously, in terms of release mechanisms, smaller particle sizes and larger specific surface areas generally lead to faster release rates; conversely, high crystallinity or the presence of surface crosslinking/shells can retard release. In the digestive environment, SNPs may undergo hydration, carrier swelling, enzymatic hydrolysis, carrier degradation, or bioactive diffusion to trigger release. The advantages of this type of nanocarrier lie in mature manufacturing methods, controllable dimensions, and ease of surface functionalization; the disadvantages include a potential propensity for aggregation and lower stability/storage capability.

### 5.2 Starch nanocrystals

Starch nanocrystals (SNCs) are nanoscale structures obtained by treating native starch granules with processes such as acid hydrolysis, enzymatic hydrolysis, or selective dissolution to remove amorphous regions or branched structures, retaining only the crystalline or highly crystalline regions (42). This results in nanostructures with extremely small dimensions and high crystallinity. In a top-down strategy, the “embedding” capacity of SNCs for active molecules may be more restricted compared to SNPs, but they offer a superior physical barrier, thereby excelling in encapsulation stability. Compared to SNPs, SNCs exhibit slower release and higher stability because the dense crystal structure restricts diffusion channels. However, encapsulation efficiency may be lower as it is more difficult for bioactive substances to penetrate the dense crystalline matrix (42). This class of structure is suitable for scenarios requiring long-term stable release or protection. Factors such as crystallinity, the ratio of residual amorphous regions, and particle size all influence the delivery performance of SNCs (principles can be referred to in Section 2.1 and 2.2).

### 5.3 Starch nanofibers

Starch-based carriers in the form of nanofibers are fabricated via spinning, electrospinning, or gel-drying methods to create filamentous structures with diameters in the nano/micro range and relatively long lengths (117). The fiber structure can encapsulate bioactive molecules by trapping them within the fiber network or coating them along the fiber surface. If formed into non-woven mats or meshes, they create a three-dimensional scaffold that immobilizes active molecules within the carrier or along the fiber surface, where the high aspect ratio of nanofibers enhances the contact probability between the carrier and the bioactive (118). Although there are fewer reports on starch nanofibers for bioactive delivery compared to particles (51), they are recognized as a viable carrier type. Because starch nanofibers have a high aspect ratio, the release rate depends on the direction of molecular diffusion. Although diffusion across the fiber thickness is short, transport along the fiber length and through the entangled fiber network is more hindered, which could result in an overall slower release. Furthermore, various operating parameters during the preparation process can be used to control the diameter to meet different release requirements (103).

### 5.4 Starch nanogels

Starch nanogels refer to colloidal nanoparticles with a hydrated, three-dimensional network structure formed by starch or its composites via non-covalent interactions or crosslinking. The network absorbs water and swells to form a hydrogel structure, distributing bioactive substances within the network pores or binding them to the polymer chains, making the system sensitive to external stimuli. Release is typically dominated by diffusion, but can also be triggered by network swelling/contraction (squeezing), network degradation, ligand exchange with the bioactive, or environmental stimuli (119). Influencing factors include network crosslinking density, pore size, hydration capacity, network degradation rate, and bioactive-network interactions. The advantages of nanogels include high loading capacity and flexible release design. Their disadvantages, however, lie in structural stability, potentially complex behavior within actual food matrices, and relatively limited research regarding preparation and scalable application.

### 5.5 V-type inclusion complexes and cyclodextrins

The term V-type helix refers to the single or left-handed helical structure formed by amylose in starch. Upon complexation with small-molecule guests (e.g., fatty acids, vitamins, or flavor compounds), it forms a highly crystalline or semi-crystalline inclusion structure known as a V-type complex (120). Cyclodextrins (CDs) are, in essence, cyclic oligosaccharides (containing 6–8 glucose units per ring, such as α, β, and γ-CD) generated from starch via enzymatic conversion. In delivery systems, cyclodextrins are frequently employed to encapsulate small-molecule bioactives via “host-guest” inclusion, thereby improving water solubility, stability, and bioavailability. Due to the finite dimensions of the inclusion cavity, this type of carrier imposes specific requirements on molecular size (102). Release typically relies on a “de-inclusion” mechanism, triggered by factors such as heat, pH, or the presence of competitive molecules. This approach is well-suited for enhancing the solubility and stability of hydrophobic molecules via inclusion. Its advantages include a simple structure, high encapsulation efficiency, and suitability for oil-soluble molecules; disadvantages include potentially low loading capacity, limited options for release modulation, and inapplicability to large macromolecules.

In summary, the inherent differences in composition, morphology, and interfacial characteristics exhibited by various starch-based nanostructures result in distinct performance combinations in encapsulation capacity, bioactive embedding modes, diffusion path lengths, enzymatic stability, and stimuli-responsiveness. Differentiating and comparing these types enables researchers to comprehend the origins of encapsulation efficiency and release patterns at the structural level, while also supporting the targeted selection of carriers for practical applications in food and nutritional systems.

## 6 Interplay among factors governing starch-based nanocarrier performance

The preceding sections discuss amylose/amylopectin ratio, crystallinity, composite effects, preparation methods, colloidal properties, and carrier type separately. However, in starch-based nanocarrier systems, these factors do not act independently. Instead, they are closely connected and jointly determine the final carrier performance. Among them, preparation methods provide the route by which the system is formed, amylose/amylopectin ratio, crystallinity, and composite effects define the material basis of the carrier, colloidal properties describe how the system behaves in dispersion, and carrier type represents the architectural outcome of their combined effects.

Preparation methods act as the primary driver because they determine how starch chains are reorganized during carrier formation, how much ordered or amorphous structure is retained, how non-starch components are incorporated, and how particles are generated and stabilized in dispersion. In this way, processing does not simply produce a carrier of a given size; it simultaneously establishes the material organization and colloidal state that later define encapsulation and release behavior. For example, nanoprecipitation has been reported to reduce relative crystallinity and increase water absorption in starch nanoparticles, indicating that the same processing step can alter both internal structure and hydration-related behavior (55). Likewise, enzymatic debranching followed by retrogradation or recrystallization promotes the formation of more ordered B-type or V-type structures, showing that processing can directly redirect the structural pathway of the carrier (114, 121). Preparation methods also determine how non-starch additives are integrated into the matrix. In glutinous rice starch systems, pressure-assisted processing promoted intimate integration between porous starch and casein, producing uniform microgels with high encapsulation efficiency and sustained release, which illustrates how processing route, composite organization, and carrier type can be established together (122). Similarly, electrostatic or covalent assembly with lysozyme or chitosan hydrochloride generates compact networked carriers rather than simple particles, again showing that processing governs not only particle formation but also the resulting carrier architecture (65, 123).

Within this processing-defined framework, amylose/amylopectin ratio and crystallinity act together to determine how starch can organize and what kind of carrier can be stabilized. A higher amylose content generally favors helical association and inclusion complex formation, whereas amylopectin-rich systems tend to exhibit more branched and compact crystalline organization because of their double-helical lamellae (48, 54). Their importance, however, lies not simply in composition itself, but in how composition is translated into structural order during processing. Once crystallinity is altered, downstream effects appear at multiple levels. Higher crystallinity generally creates denser packing and stronger diffusion barriers, whereas lower crystallinity leads to a more open and hydrated structure, which favors faster water ingress and release (55). These structural states also influence colloidal behavior, because compactness, hydration, and aggregation tendency affect how particles remain dispersed and how stable they are during storage or digestion. At the same time, they constrain carrier type. V-type inclusion systems rely on amylose helicity and suitable guest accommodation, whereas more amorphous reorganized systems are more compatible with particulate carriers that depend on surface area and diffusion path rather than inclusion geometry. In this sense, amylose/amylopectin ratio does not act directly on performance as an isolated variable, but through its interaction with crystallinity, colloidal behavior, and the carrier form that eventually emerges.

Composite effects add another level of interaction because non-starch additives can change structural order, colloidal stability, and carrier architecture at the same time. The most useful examples are not those that compare unrelated systems, but those showing several linked changes within the same formulation. In curcumin-loaded starch systems, *Mesona chinensis* polysaccharide inhibited aggregation while decreasing structural order, and this combined change was accompanied by higher encapsulation efficiency and longer gastrointestinal release, indicating that composite effects can simultaneously modify structure, colloidal expression, and final functionality (114). Sodium caseinate provides another strong example. In OSA starch systems, it increased surface charge and promoted formation of a denser network or more compact nanoparticles, which improved encapsulation efficiency and loading capacity (115, 116). In contrast, choline chloride–lactic acid reduced particle size but also disrupted crystalline structure and weakened starch–curcumin association, leading to lower encapsulation efficiency and loading capacity (124). These examples are important because they show that a seemingly favorable change in one parameter, such as smaller particle size, does not necessarily improve performance if it is accompanied by unfavorable changes in crystallinity or carrier–bioactive affinity. Thus, composite effects cannot be treated as simple add-ons; they reshape the relationship between structure, colloidal behavior, and final performance.

Colloidal properties are therefore best understood as the integrated manifestation of the preceding material and processing effects. Particle size, PDI, and *ζ*-potential reflect how successfully the carrier has been formed, stabilized, and organized, but their functional meaning depends on the structural and compositional context in which they arise. This is why carrier type becomes important at the final stage of the interaction chain. Carrier type is the architectural expression of how processing, structural organization, composite effects, and colloidal stabilization have come together in a given system. Nanoparticles, nanocrystals, nanogels, V-type inclusion complexes, and composite matrices all embody different combinations of these upstream factors, and once formed, they determine which mechanisms dominate performance. In particulate carriers, particle size distribution and *ζ*-potential become especially important because interfacial area and dispersion stability strongly affect encapsulation and release. In nanogels, crosslinking density, hydration, and swelling become central because release depends on network relaxation and diffusion through a three-dimensional matrix. In inclusion-type carriers, amylose helicity and guest fit become decisive because retention and release depend on inclusion behavior.

**Figure 3:**
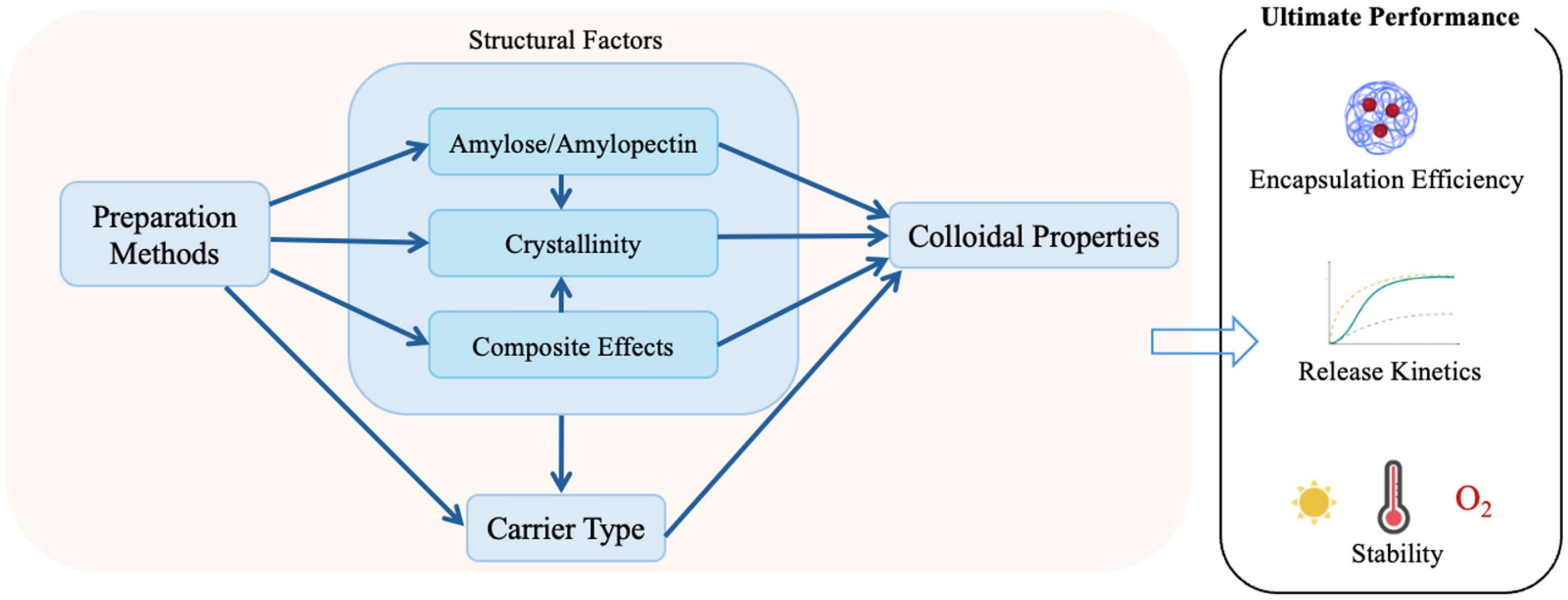
Interplay among factors governing starch-based nanocarrier performance.

Overall, starch-based nanocarrier performance should therefore be understood as the outcome of a connected system in which preparation methods establish material and colloidal states, these states give rise to a specific carrier type, and the resulting architecture governs how encapsulation, protection, and release are ultimately achieved (Figure 3).

## 7 Conclusions and perspectives

**Figure 4:**
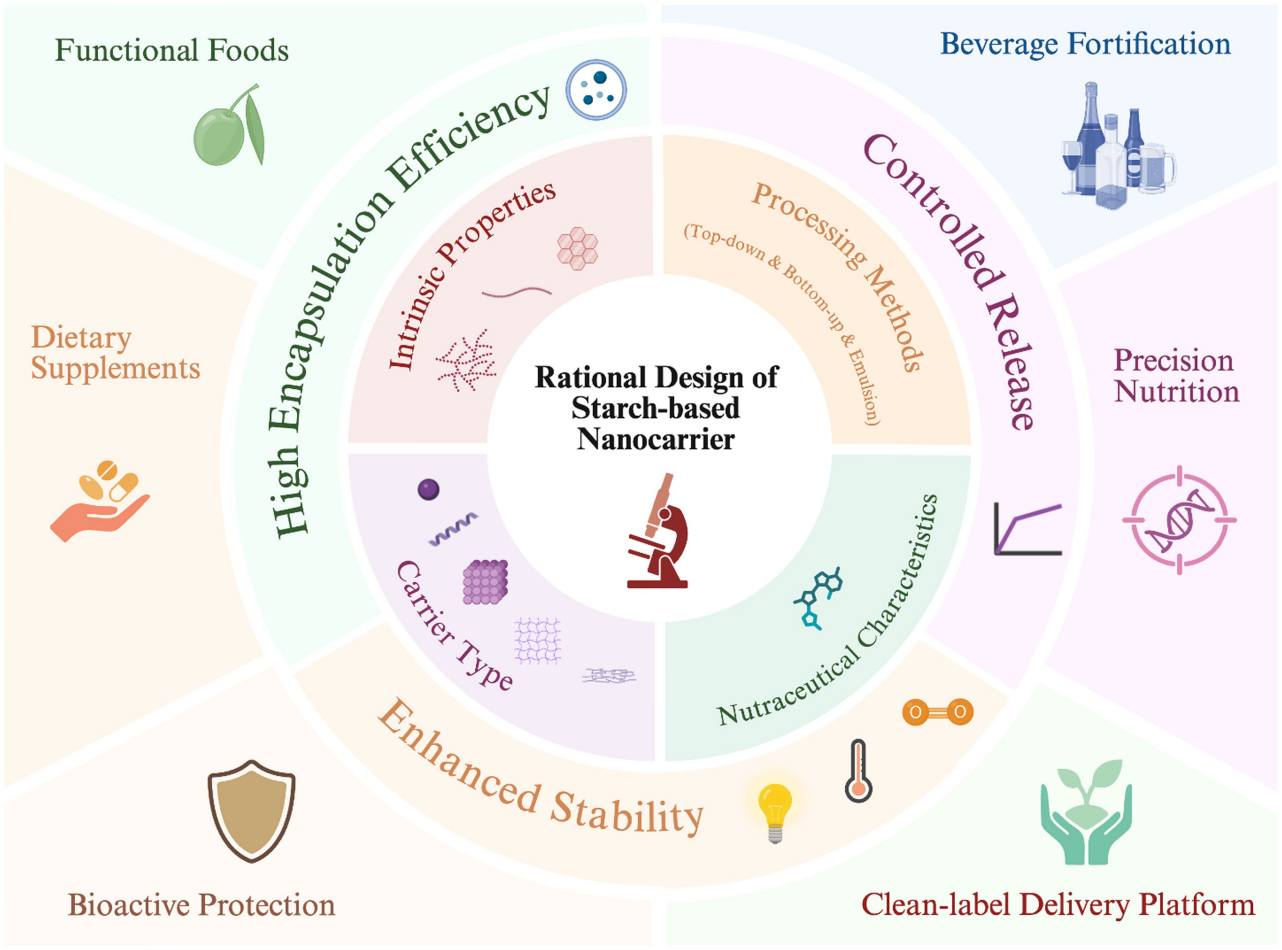
Starch-based nutraceutical delivery systems: from rational design and property control to food applications.

This review has systematically summarized the key factors influencing the performance of starch-based nanocarriers for nutraceuticals delivery, ranging from intrinsic material attributes and preparation methods to the properties of the encapsulated active compounds. Our work fills a critical gap by clarifying the underlying physicochemical mechanisms that drive encapsulation efficiency and release kinetics. The discussion reveals that successful delivery is not dependent on a single factor, but on the synergistic interplay among colloidal properties, material structure, processing-induced organization, and cargo-specific properties. These factors collectively regulate carrier–bioactive interactions, structural stability, molecular diffusion pathways, and enzymatic accessibility, thereby determining encapsulation capacity and release behavior. Importantly, these relationships can be generalized into a unified factor–property–function framework, in which formulation and processing variables define structural and interfacial properties, which in turn govern molecular interactions and mass transfer processes, ultimately determining delivery performance. From an application perspective, these insights enable the rational selection and design of starch-based nanocarriers tailored to specific nutraceuticals, such as improving the stability of bioactive compounds, enhancing gastrointestinal bioaccessibility, or achieving targeted and sustained release profiles. This provides practical guidance for translating laboratory-scale formulations into functional food and nutraceutical products (Figure 4).

Looking to the future, the primary challenge remains the complexity of optimizing these multi-dimensional factors for industrial-scale applications. In this context, Design of Experiments (DoE) provides a systematic framework to capture interactions among formulation and processing variables. For instance, in curcumin-loaded starch carriers, the combined variation of crosslinker, surfactant, and stirring rate was shown to simultaneously regulate encapsulation efficiency and release behavior, with responses accurately predicted by response surface models (125). Similarly, in starch nanoparticles loaded with phenolic compounds from propolis extract, starch concentration, anti-solvent ratio, and mixing rate jointly controlled particle size and adsorption efficiency, yielding optimized nanosystems with predictable performance (126). Beyond delivery performance, DoE has also been applied to starch nanoparticle design, where interacting processing variables such as ultrasonic power and treatment time, pressure conditions, and solvent composition were shown to jointly influence particle size, crystallinity, and colloidal stability, with strong agreement between predicted and experimental outcome (127–129). These studies collectively demonstrate that starch-based nanocarriers behave as predictable systems governed by interacting parameters rather than independent variables. Building on this foundation, the integration of machine learning offers a further transformative opportunity. DoE-generated datasets provide structured inputs for data-driven models, enabling the identification of hidden patterns and the prediction of optimal formulation conditions for specific encapsulation and release targets. By combining systematic experimental design with artificial intelligence, future research can transition from empirical formulation toward predictive and precision engineering, ultimately accelerating the development of robust, high-performance starch-based delivery systems for the functional food industry.
